# Open-source autosampler for elemental and isotopic analyses of solids

**DOI:** 10.1016/j.ohx.2020.e00123

**Published:** 2020-07-10

**Authors:** Matheus C. Carvalho, William Eickhoff, Michael Drexl

**Affiliations:** a Centre for Coastal Biogeochemistry Research. School of Environment, Science and Engineering. Southern Cross University. PO Box 157, Lismore 2480, NSW, Australia; b Southern Cross Plant Science. Southern Cross University. PO Box 157, Lismore 2480, NSW, Australia; c Southern Cross Geoscience. Southern Cross University. PO Box 157, Lismore 2480, NSW, Australia

**Keywords:** 3D printing, Autosampler, Arduino, AutoIt, Carbon, Elemental analysis, Laboratory automation, Nitrogen, OpenSCAD, Stable isotopes, Sampling

## Abstract

Elemental and isotopic analyses are performed using elemental analyzers, and are widely employed for diverse scientific fields. An elemental analyzer is typically equipped with an autosampler. Here we present an open-source autosampler for elemental and isotopic analysis of solid samples. The autosampler consists of 1) a sampling table, on which a carousel pushes samples inside an orifice, and 2) a purging pipe, placed directly beneath the orifice, where the sample is purged off surrounding air, and then delivered to the reaction tube. The action of the purging pipe ensured that air contamination, an issue for the analysis of some elements like nitrogen and oxygen, was negligible, and results for elemental and isotopic composition of nitrogen and carbon were inside specs. Compared to commercial alternatives, the autosampler presented here has the advantages of lower cost to build and maintain, universal compatibility with instruments from different manufacturers, capacity do deal with bulky samples, capacity for a larger number of samples in a single run, and no necessity for time-consuming purging in-between sample loading. The autosampler could potentially also be employed for the analyses of other elements (e.g. oxygen, hydrogen and sulfur) because they are performed using similar equipment.

## Graphical abstract

**Figure:**
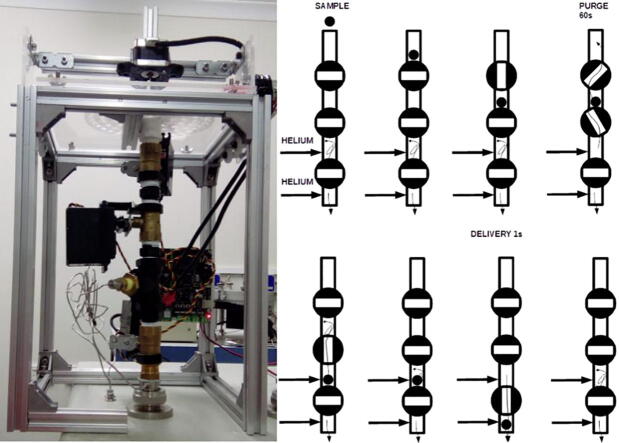

## 1 Hardware in context

Specifications table

**Table:** 

Hardware name	Oseas
Subject area	• Chemistry and Biochemistry • Environmental, Planetary and Agricultural Sciences • Medicine, Forensics
Hardware type	• Solid sample handling
Open Source License	GNU General Public License (GPL) 3.0
Cost of Hardware	AU$ 542 plus extras
Source File Repository	https://osf.io/utzse/ or https://dx.doi.org/10.17605/OSF.IO/UTZSE

Elemental analysis is one of the most fundamental chemical measurements. Of special significance for environmental and ecological research are the analyses of carbon and nitrogen amounts and isotopic compositions in bulk samples, which are carried using elemental analyzers operating on variations of the Dumas principle [1], [2]. These analyzes used to be laborious and time consuming, but became very popular after automation, which allowed rapid throughput and easiness of operation [3], [4], [2]. Since then, these measurements have been performed in numerous environmental and geochemical studies covering all sorts of environments [5], [6], [7]. They have also been employed for forensic investigations, including drug testing, food provenancing, and crime solving [8].

Elemental analyses of carbon and nitrogen are carried out using elemental analyzers. Autosamplers are a standard part of most elemental analyzers, and some accessories for these autosamplers have been developed by scientists, either to deal with special analytical needs [9], or to make operation easier [10]. Autosamplers themselves are also among the most accessible scientific devices in terms of fabrication by non-specialized engineers, with several examples available in the literature [11], [12], [13], [14], [15], [16], [17], [18], [19], [20]. While in some cases these autosamplers have been integrated to other in-house made components [17], [16], [13], [18], [19], the integration of in-house made autosamplers to commercial, off-the-shelf equipment like elemental analyzers has been a difficult challenge for several decades [21], [22], [23], only recently facilitated by the adoption of AutoIt for laboratory automation.

AutoIt is a scripting language for the Windows operating system that allows easy integration of analytical devices [24], [25], [26], [27]. By means of AutoIt, open-source autosamplers for a range of analytical purposes have been presented, covering both fluid (liquids and gases) [28], [29] and solid samples [30]. However, none of these autosamplers is appropriate to handle solid samples for elemental analysis, because in these devices the samples must be introduced into a reactor without air contamination. Here we present an open-source autosampler with the necessary characteristics for elemental analysis that can fully replace commercial models for a comparatively small cost (AU$540 plus extras).

## 2 Hardware description

**Fig. 1:**
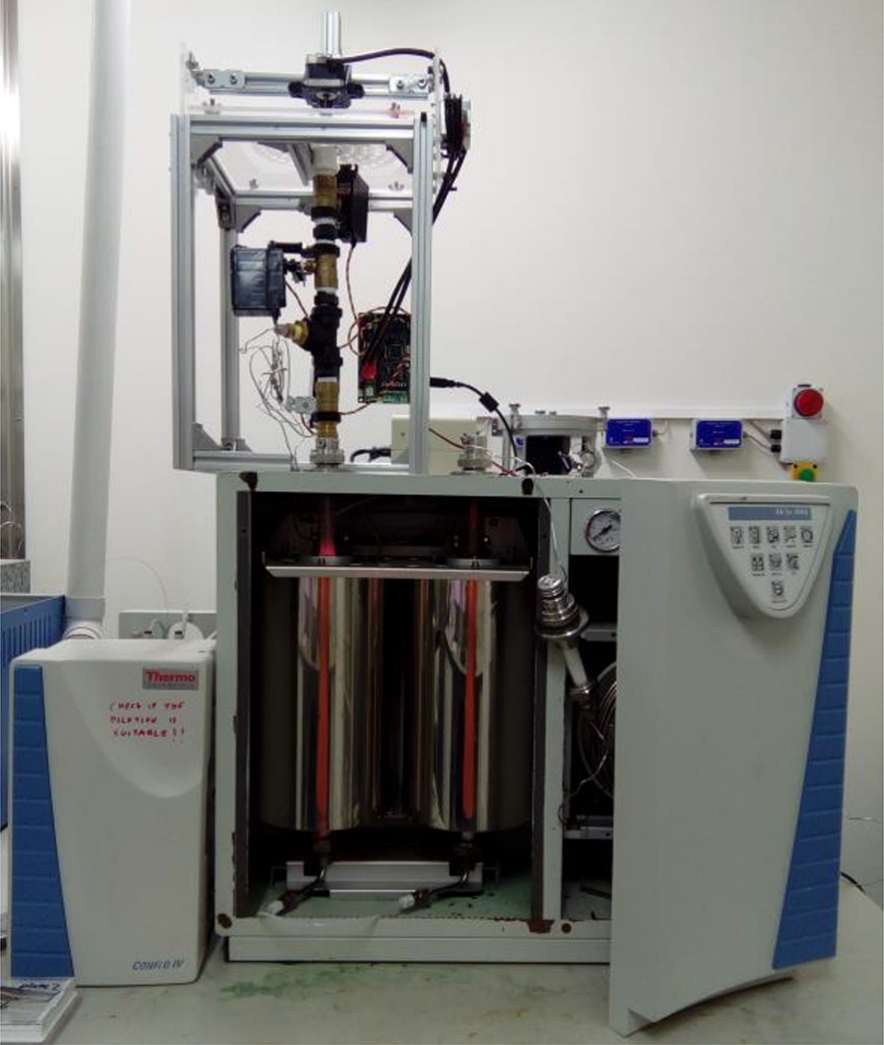
EA autosampler mounted on top of an elemental analyzer. The sample table is the four-legged acrylic sheet supporting the carousel. The purging pipe is the pipe beneath the sampling table and connected to the elemental analyzer.

**Fig. 2:**
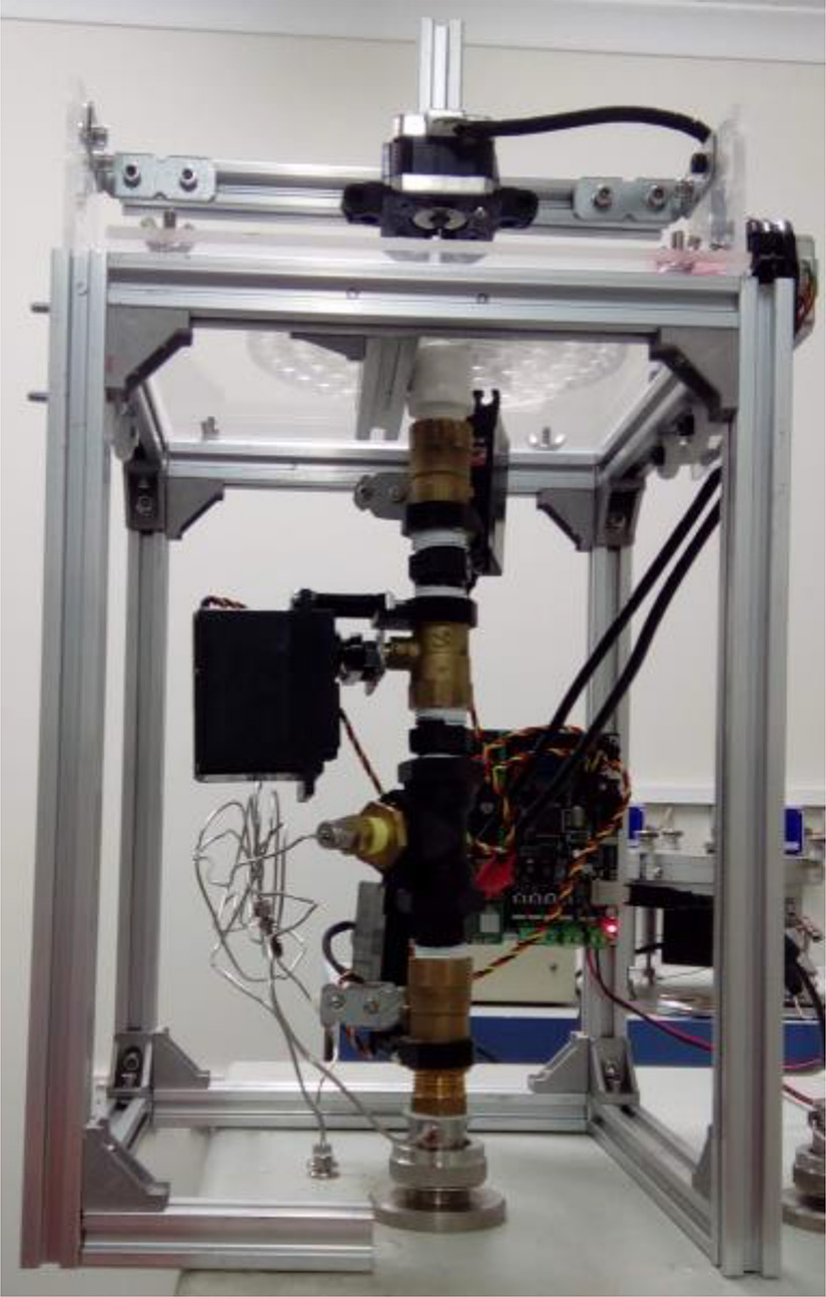
EA autosampler. A table made of aluminum extrusion profiles holds an acrylic sheet, on top of which a carousel spins delivering samples through a hole on the sheet. The hole is placed directly above the purging pipe, where three ball valves, actuated by servo motors, ensure that minimal air contamination occurs when the sample is delivered to the elemental analyzer.

The autosampler described here consists of two parts: 1) a sampling table and 2) a purging pipe (Fig. 1). The sampling table consists of a table on which a carousel is placed (Fig. 2). The carousel contains five concentric circles of holes. There are two stepper motors: one spins the carousel, while the other pushes a gantry holding the carousel forward so that the inner circles containing samples can also spin over the orifice, delivering the samples.

The purging pipe consists of 3 ball valves connected in series, each controlled by a servo motor (Fig. 2). All valves and connectors in the setup are half inch in diameter, except those from the pipe to helium supply, which need to be reduced to 1/8 or 1/16 in. diameters, depending on the instrument’s specifications. The topmost part of the pipe is simply a straight pipe or connector to minimize the space between the orifice of the plate holding the carousel and the topmost valve. Just beneath are the top and middle valves, which are interconnected using a simple connector. The space between the two valves is called “purging chamber”. The middle valve is connected to the bottom valve using a T piece, which is connected to a carrier gas line. The space between the middle and bottom valve is called “isolation chamber”. The bottom valve is connected to the elemental analyzer using another T piece, which is also connected to a carrier gas supply.

**Fig. 3:**
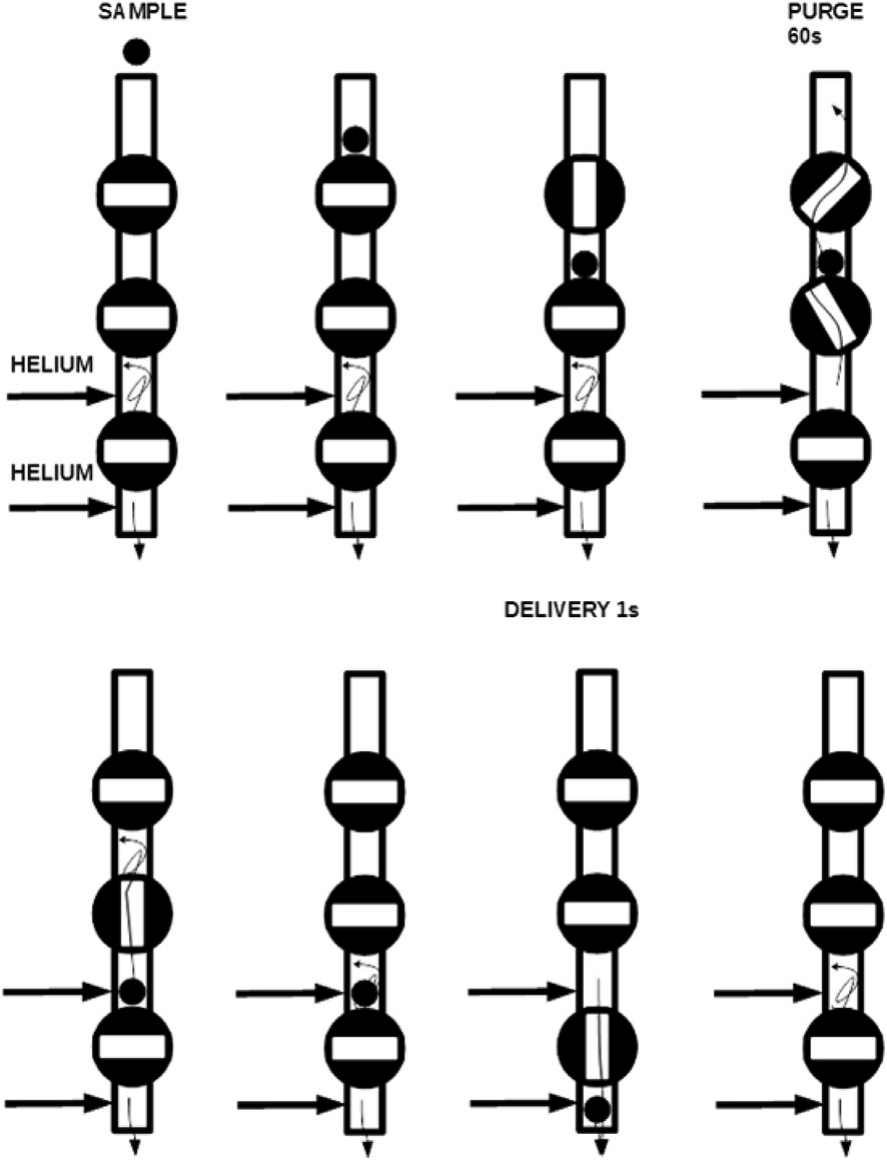
Scheme of the operation of the purging pipe. Arrows indicate helium flow. Full description in the text, Section 2.

The purging pipe ensures that a minimal amount of air is carried along the sample for analysis (Fig. 3). The sample falls on top of the top valve, which is opened and partially closed after the sample drops through it. Then the middle valve is partially opened, such that carrier gas purges the purging chamber. After a certain time (typically 1 min), the top valve is closed, and the middle valve is fully opened, so that the sample falls on top of the bottom valve, inside the isolation chamber. The middle valve is fully closed, and then the bottom valve is fully opened, allowing the sample inside the elemental analyzer. The bottom valve is then immediately fully closed, thus minimizing air contamination.

Both stepper and servo motors are controlled by a MKS Gen-L control board, also used in other autosamplers [29], [30]. The firmware of the board is Marlin, fully open-source, enabling the use of G-code [29], [30]. The integration of the autosampler and elemental analyzer is done using AutoIt [28], [29], [30], [24].

A video showing the autosampler working is presented in Supplementary Information 1.

## 3 Design files

### 3.1 Design files summary

In addition to the hyperlinks provided for the location of the files, this DOI link is also available for the master page with all links: https://doi.org/10.17605/OSF.IO/UTZSE.

**Table:** 

Design file name	File type	Open source license	Location of the file
Carousel	OpenSCAD	GNU General Public License (GPL) 3.0	https://osf.io/vs9py/
Gantry support	Libre Office Draw	GNU General Public License (GPL) 3.0	https://osf.io/ezgcv/
Table top	Libre Office Draw	GNU General Public License (GPL) 3.0	https://osf.io/ezgcv/
NEMA 17 stepper motor mount	OpenSCAD	GNU General Public License (GPL) 3.0	https://osf.io/d64x5/
Servo to ball-valve connector	OpenSCAD	GNU General Public License (GPL) 3.0	https://osf.io/gwxkr/
Spacer for 4 mm screws	OpenSCAD	GNU General Public License (GPL) 3.0	https://osf.io/mv5ju/
Spacer for 3 mm screws	OpenSCAD	GNU General Public License (GPL) 3.0	https://osf.io/4kmwf/
Sieve for half inch pipe	OpenSCAD	GNU General Public License (GPL) 3.0	https://osf.io/qjpfu/

## 4 Bill of materials

Freight costs are not included in this table. They can be significant for bulky items, like extrusion profiles. Also, specific connections between the purging pipe and the elemental analyzer are not included, as they are likely to vary depending on the elemental analyzer model.

**Table:** 

Figure or reference	Component	Number	Cost per unit –AU$	Total cost –AU$	Source of materials	Material type
Fig. 2	T slot 20x20mm, 35 cm	4	$3.85	$14.00	https://www.ebay.com.au/itm/2020-Aluminium-Extrusion-T-Slot-Profile-Euro-Standard/283479874340	Aluminum
Fig. 2, Fig. 9	T slot 20x20mm, 22 cm	7	$2.42	$16.94	https://www.ebay.com.au/itm/2020-Aluminium-Extrusion-T-Slot-Profile-Euro-Standard/283479874340	Aluminum
Fig. 2, Fig. 12	T slot 20x20mm, 10 cm	3	$1.10	$3.30	https://www.ebay.com.au/itm/2020-Aluminium-Extrusion-T-Slot-Profile-Euro-Standard/283479874340	Aluminum
Fig. 2, Fig. 12	T slot 20x20mm, 25 cm	1	$2.75	$2.75	https://www.ebay.com.au/itm/2020-Aluminium-Extrusion-T-Slot-Profile-Euro-Standard/283479874340	Aluminum
Fig. 10	2028 bracket, pack with 20	1	$10.15	$10.15	https://www.aliexpress.com/item/32642711369.html	Aluminum
Fig. 7	Long 90°bracket	2	$1.06	$2.12	https://www.bunnings.com.au/carinya-50-x-50-x-20-x-2mm-make-a-bracket_p3960590	Steel
Fig. 2, Fig. 6, Fig. 12	NEMA 17 stepper motor	2	$13.66	$27.32	https://www.ebay.com.au/itm/Nema-17-Stepper-Motor-40Ncm-0-9A-4-Lead-90cm-Lead-Cable-for-DIY-3D-Printer-CNC/113127734830	Metal, plastic
Fig. 13	Shaft coupler, 5 mm to 8 mm	1	$5.56	$5.56	https://www.ebay.com.au/itm/5-to-8mm-Shaft-Coupler-Aluminium-Blue-Coupling-CNC-Machine-Lead-Flux-Workshop/113212021207	Metal
Fig. 2, Fig. 11	Acrylic sheet, A4 size	2	$9.50	$19.00	https://www.ebay.com.au/itm/Clear-Acrylic-Precision-Cut-Sheets-Commonly-known-as-perspex-plexiglass/322038335759	Plastic
Fig. 6, Fig. 19	M3 screws, 10 mm length, pack with 10	2	$7.20	$14.40	https://www.ebay.com.au/itm/M3-M4-M5-M6-M8-Socket-Head-Cap-Screw-Stainless-Steel-304-Metric-Coarse/142732934590	Metal
Fig. 15	M3 screws (16 mm length), pack with 10	1	$7.20	$7.20	https://www.ebay.com.au/itm/M3-M4-M5-M6-M8-Socket-Head-Cap-Screw-Stainless-Steel-304-Metric-Coarse/142732934590	Metal
Fig. 15	M3 nuts, pack with 10	1	$5.50	$5.50	https://www.ebay.com.au/itm/263624205539	Metal
Fig. 19	M3 hammer nuts, pack with 10	1	$6.50	$6.50	https://www.ebay.com.au/itm/10-x-M3-T-Slot-hammer-nuts-for-20-series-extrusion-aluminium-profile/113534394323	Metal
Fig. 16	M4 screws, 40 mm length, fully threaded, pack with 10	1	$1.87	$1.87	https://www.aliexpress.com/item/33020981797.html	Metal
Fig. 16	M4 hammer nuts, pack with 10	1	$6.00	$6.00	https://www.ebay.com.au/itm/10-x-2020-M4-T-Slot-hammer-nuts/113218156075	Metal
Fig. 16	M4 nuts, pack with 10	1	$5.50	$5.50	https://www.ebay.com.au/itm/263624205539	Metal
Fig. 7, Fig. 8, Fig. 9, Fig. 10, Fig. 12	M5 screws, 10 mm length, pack with 100	1	$21.00	$21.00	https://www.ebay.com.au/itm/M3-M4-M5-M6-M8-Socket-Head-Cap-Screw-Stainless-Steel-304-Metric-Coarse/142732934590	Metal
Fig. 7, Fig. 8, Fig. 9, Fig. 10, Fig. 12	Washer for M5 screw, pack with 100	1	$9.80	$9.80	https://www.ebay.com.au/itm/142552908688	Metal
Fig. 5, Fig. 7, Fig. 8, Fig. 9, Fig. 10, Fig. 12	Hammer nut for M5 screw, pack with 100	2	$9.69	$19.38	https://www.ebay.com.au/itm/50x-Hammer-Head-T-Nut-Drop-In-M5-for-30-Series-European-Aluminum-Slot/272532621625	Metal
Fig. 11	M5 screws with hammer-head, 16 mm length, pack with 10	1	$3.18	$3.18	https://www.ebay.com.au/itm/DIY-M5-Hammer-Head-T-Bolt-Screw-For-20Series-Aluminum-T-slot-10mm-12mm16mm-25mm/312480457816	Metal
Fig. 11	Wingnut for M5 screw, pack with 5	1	$8.90	$8.90	https://www.ebay.com.au/itm/263678710533	Metal
Fig. 5	M5 screws, 35 mm length, fully threaded pack with 10	1	$3.36	$3.36	https://www.aliexpress.com/item/32868905756.html	Metal
Fig. 5	Spacer for M5 screws, 10 mm length, set with 25	1	$2.47	$2.47	https://www.aliexpress.com/item/25pcs-White-Plastic-Nylon-ABS-Round-Non-Threaded-Column-Standoff-Support-Spacer-Washer-For-M5-Screw/32650018699.html	Nylon
Fig. 6, Fig. 9	GT2 Timing belt, 1 m	1	$1.83	$1.83	https://www.ebay.com.au/itm/1X-Courroie-2GT-6mm-timing-belt-3D-printer-courroie-GT2-1M-S3M8/233200843536	Rubber
Fig. 6, Fig. 9	20 teeth Pulley for GT2 timing belt, 20 teeth	1	$4.50	$4.50	https://www.ebay.com.au/itm/LearCNC-GT2-Timing-Belt-Pulley-RepRap-Prusa-RAMPS-Kossel-3D-Printer-Nema17/201145021195	Metal
Fig. 6, Fig. 9	Wheels 625ZZ, 21.5 cm in outer diameter, pack with 9	1	$7.91	$7.91	https://www.aliexpress.com/item/9pcs-3D-Printer-Kossel-Nylon-Plastic-Wheel-with-Bearings-Bearing-Roller-Wheel-POM-5-x-21/32816554334.html	Metal, plastic
Fig. 4, Section 3	3D-printed carousel	1	<$0.10	<$0.10	Section 3.1	PLA
Fig. 16, Section 3	3D-printed servo to ball valve connector	3	<$0.10	<$0.30	Section 3.1	PLA
Fig. 16, Section 3	3D-printed spacer for M4 screws	6	<$0.05	<$0.30	Section 3.1	PLA
Fig. 12, Section 3	3D-printed NEMA 17 motor mount	1	<$0.10	<$0.10	Section 3.1	PLA
Fig. 18, Section 3	3D-printed sieve for half inch pipe	1	<$0.10	<$0.10	Section 3.1	PLA
Fig. 19	MKS Gen L control board with stepper motor drivers (A4988)	1	$32.01	$32.01	https://www.ebay.com.au/itm/Part-A4988-MKS-Gen-L-V1-0-Integrated-Mainboard-Control-Board-Motherboard/123147319056	Metal, plastic
Fig. 20	Cables with adapters to connect stepper motors to control board (1 m), set with 4	1	$3.75	$3.75	https://www.ebay.com.au/itm/4x-6-pin-JST-to-4-pin-Dupont-Connector-for-Nema17-Stepper-Motor-Cable-3D-Printer/183199926610	Metal, plastic
Fig. 20	Cable wrapper 2 m	1	$8.89	$8.89	https://www.ebay.com.au/itm/2M-Spiral-Cable-Wrap-Tidy-Cord-Wire-Banding-Storage-Organizer-10mm-25mm-3-Colors/293034936624	Plastic
Fig. 20	USB cable, 5 m long	1	$6.50	$6.50	https://www.ebay.com.au/itm/USB-2–0-Type-A-Male-to-B-Printer-Cable-for-HP-Canon-Dell-Brother-Epson-Xerox/111656844488	Metal, plastic
Not shown	24 V, 5.62A power supply	1	$29.00	$29.00	https://www.ebay.com.au/itm/AU-Power-Supply-Adapter-Transformer-AC240V-To-DC24V-1–2-3–4-5A-for-LED-Strip/202120736297	Metal, plastic
Not shown	Adapter for power supply to the control board (2.1 mm illuminated polarity sensing DC socket)	1	$8	$8	https://www.ebay.com.au/itm/NEW-2–1mm-Illuminated-Polarity-Sensing-DC-Socket-WQ7289/252988850556	Metal, plastic
Fig. 2, Fig. 16	Hitec HS-805BB servo motor	3	$60.00	$180.00	https://www.modelflight.com.au/hitec-hs-805bb-mega-quarter-scale-indirect-drive-dual-ball-bearing.html	Metal, plastic
Fig. 14	½ inch ball valve	3	$7.70	$22.10	https://www.bunnings.com.au/kinetic-15mm-brass-ball-valve_p4790274	Brass
Fig. 18	½ inch female-female-female T tube	1	$4.50	$4.50	https://www.bunnings.com.au/garden-rain-0–5-poly-irrigation-f-f-f-tee_p3100169	Plastic
Fig. 18	½ inch male-male connector	4	$1.15	$4.60	https://www.bunnings.com.au/garden-rain-0–5-poly-irrigation-nipple_p3100149	Brass or plastic
Fig. 2	½ inch unthreaded to threaded connector	1	$0.95	$0.95	https://www.bunnings.com.au/holman-15mm-x-1-2-pvc-valve-socket_p3141883	PVC
Fig. 18	Thread tape (roll)	1	$3.00	$3.00	https://www.bunnings.com.au/gastite-12mm-x-10m-yellow-threadseal_p4920513	Teflon
Not shown	O-ring set	1	$3.85	$3.85	https://www.bunnings.com.au/kinetic-assorted-sizes-o-ring-kit-43-pack_p4920305	Rubber
Fig. 14	Perforated steel stripping (20 × 2 × 0.2 cm)	2	$1.72	$3.44	https://www.bunnings.com.au/carinya-20-x-200-x-2mm-flat-make-a-bracket_p3960600	Steel

## 5 Build instructions

### 5.1 Custom parts

Acrylic sheets need be cut and drilled as per provided designs (Section 3).

**Fig. 4:**
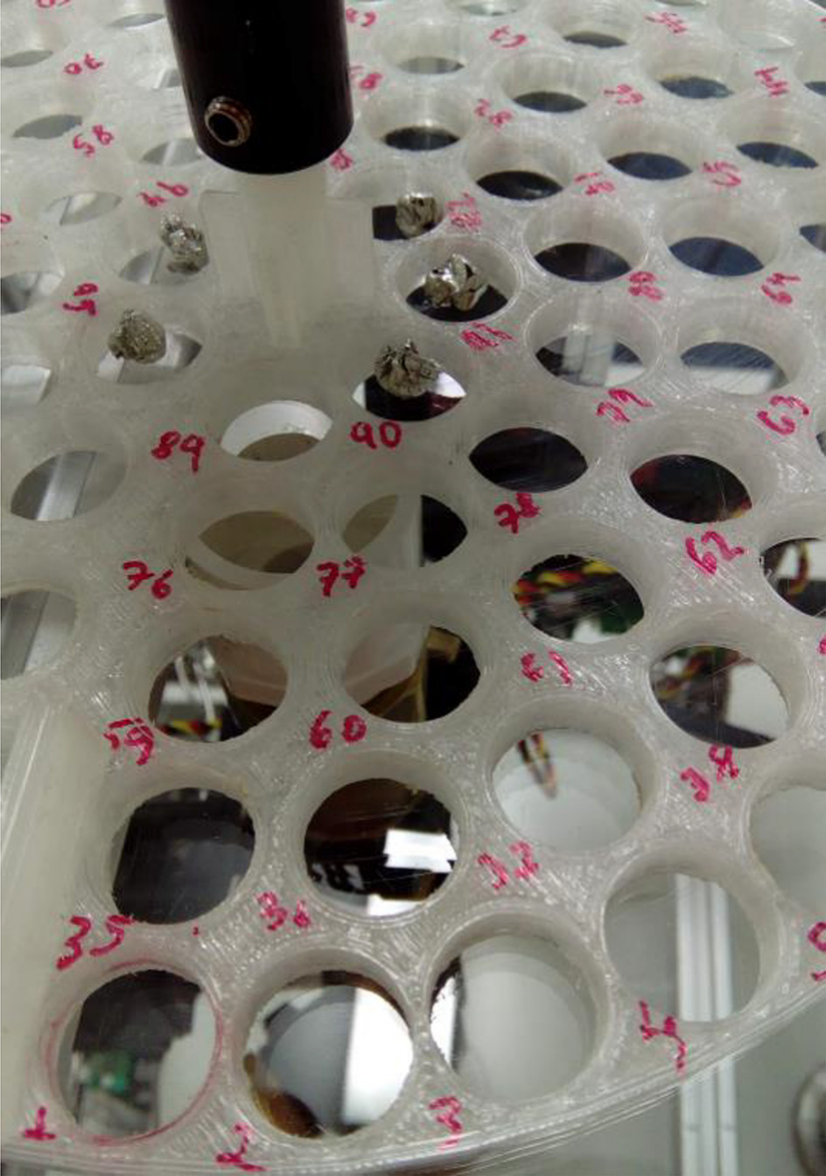
Carousel made of clear PLA, allowing writing of the sample positions with a pen.

3D-printed parts should be printed with at least 75% infill, except the carousel, for which 20% suffices. It is recommended that the carousel is printed using clear plastic so that the holes can be easily numbered using a pen or a marker (Fig. 4).

### 5.2 Assembling the sampling table

**Fig. 5:**
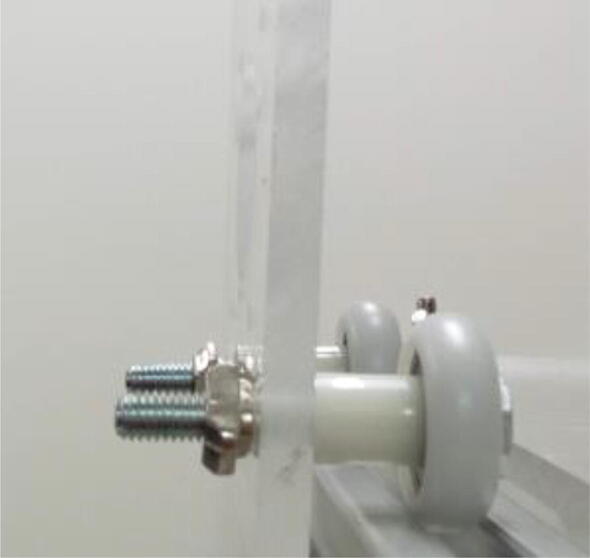
Wheels fixed to the one of the acrylic supports.

Fix the wheels to the acrylic supports (Fig. 5). First place a wheel (625ZZ, 21.5 cm in outer diameter) around an M5, 35 mm screw, followed by a 10 mm nylon spacer. Attach the screw to the acrylic plate and fix it using an M5 hammer nut. Do this for 4 screws for each of the two plates.

**Fig. 6:**
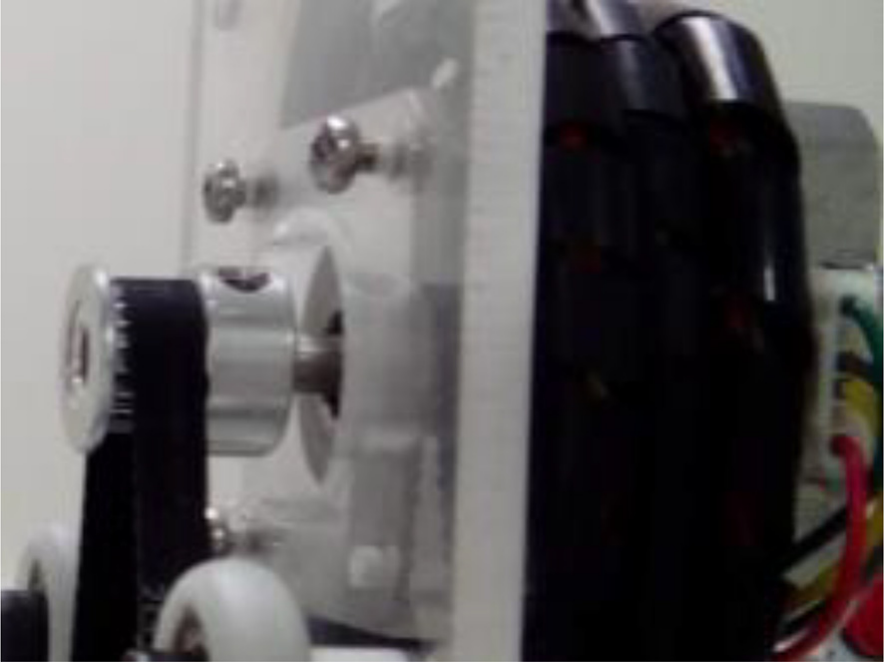
Stepper motor fixed to one of the acrylic supports. Here only 3 screws are holding the motor.

Fix one stepper motor to one of the acrylic supports using M3 screws (Fig. 6).

**Fig. 7:**
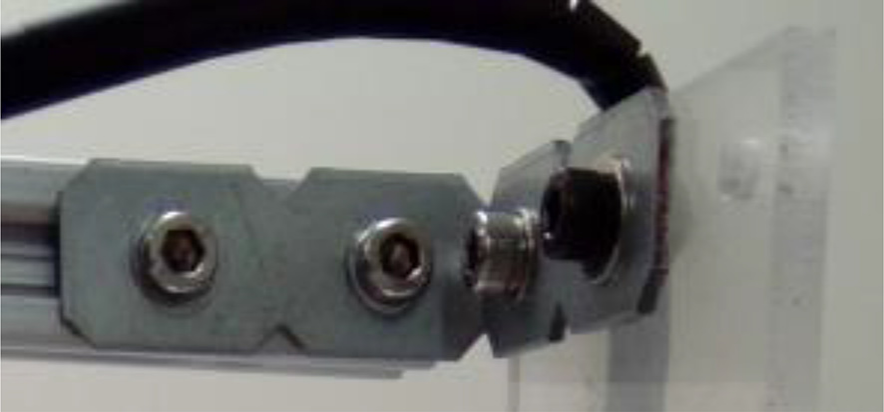
Bracket fixed to acrylic support.

Fix long brackets to both acrylic supports using M5 screws, spacers and hammer nuts (Fig. 7). Do not fix the brackets to the extrusion profile yet.

**Fig. 8:**
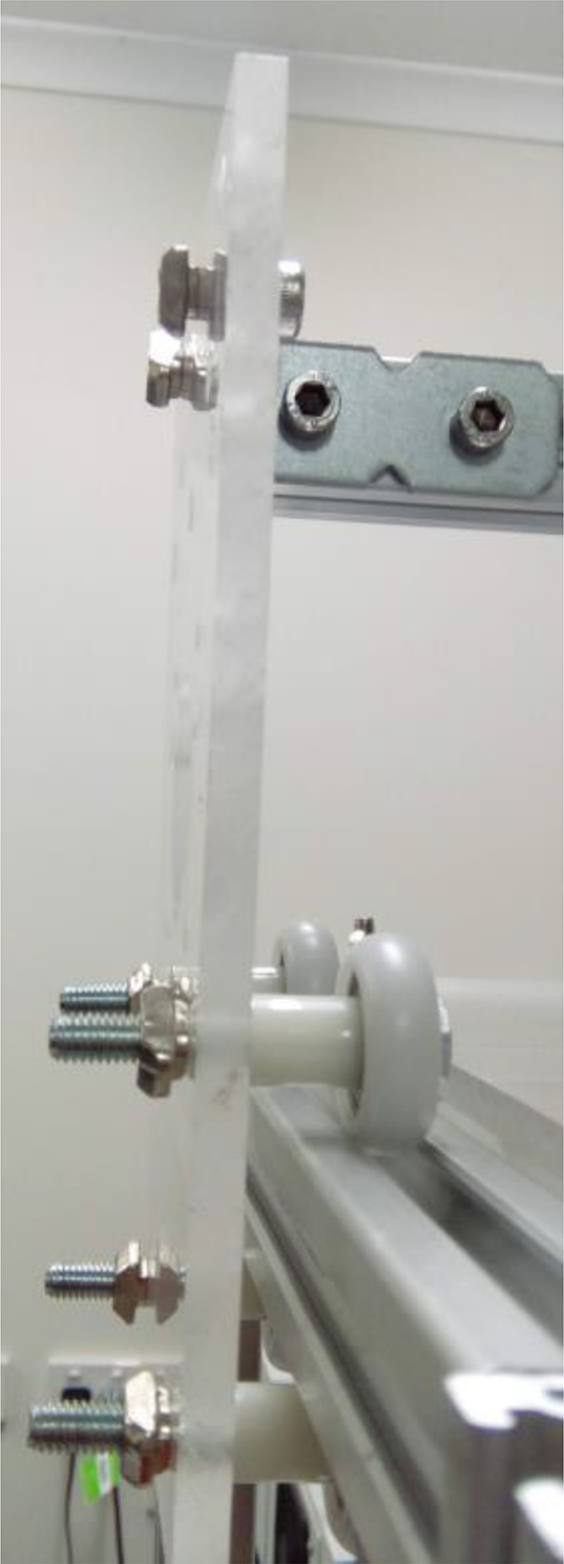
Acrylic support placed on a 22 cm aluminum extrusion profile. The support should move smoothly with the wheels inside the groove.

Slide each acrylic sheet already holding its wheels on a 22 cm t slot (Fig. 8).

**Fig. 9:**
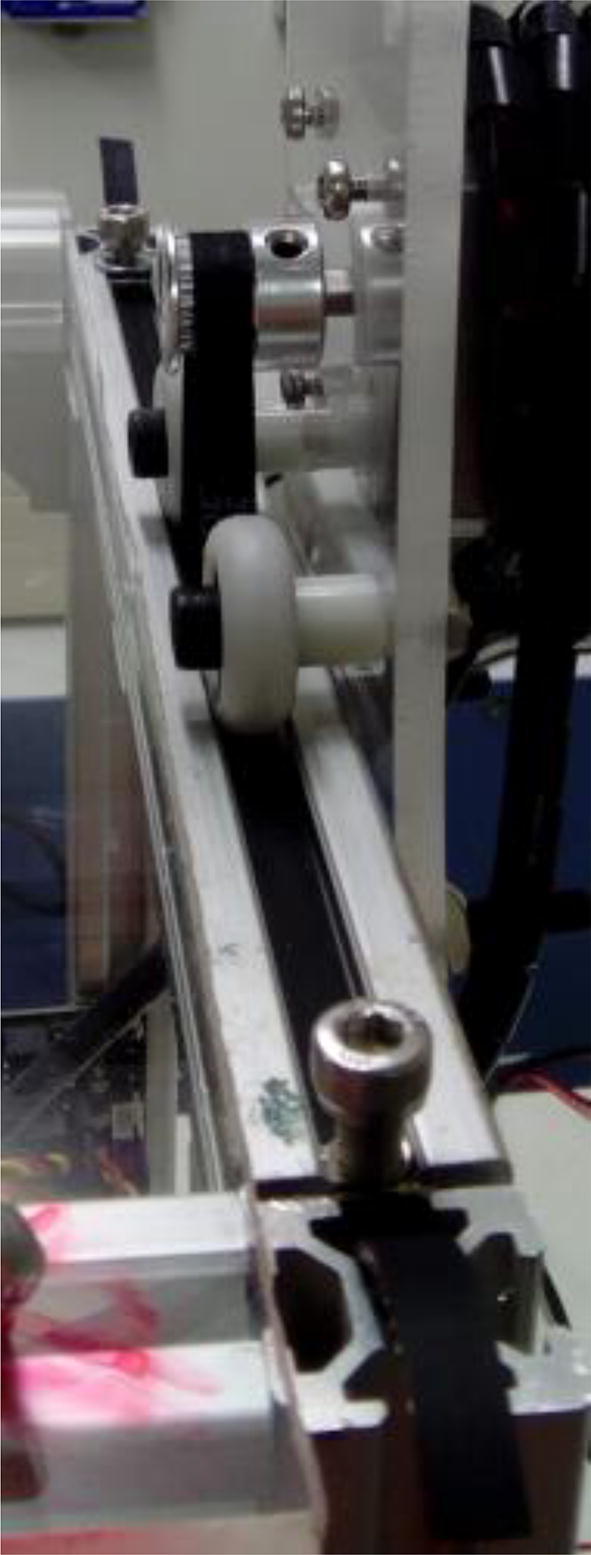
Timing belt around stepper motor pulley, and fixed on aluminum extrusion profile.

Fix pulley on stepper motor, place timing belt around pulley, and fix it on t slot (Fig. 9). Use M5, 10 mm screws and hammer nuts to fix the belt to the profile. Align the pulley teeth to the groove on the extrusion profile.

**Fig. 10:**
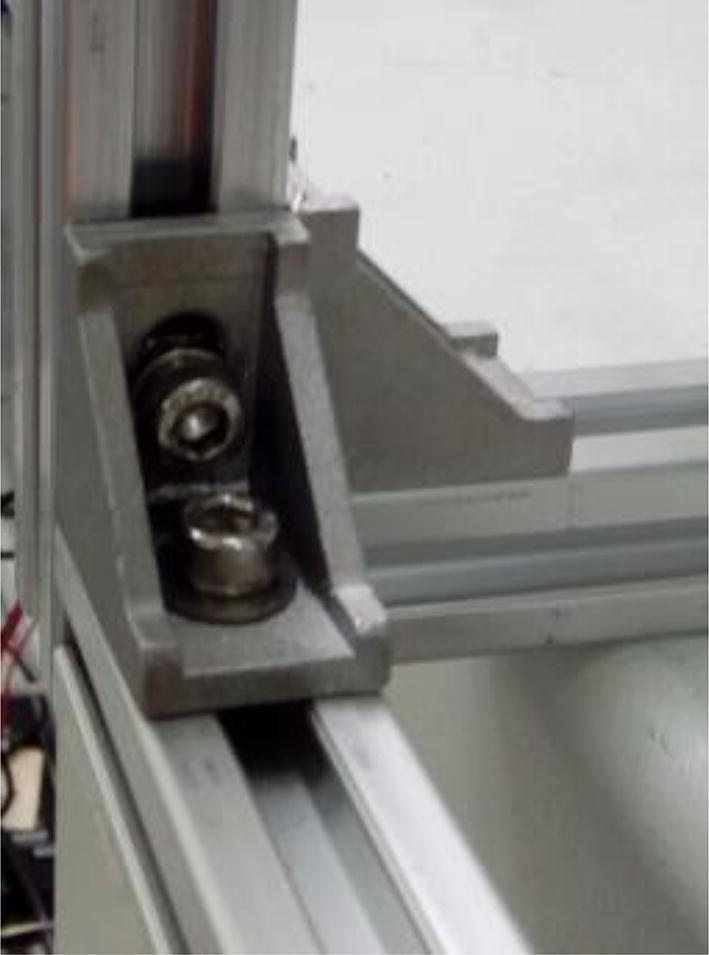
Bracket (2028) connecting extrusion profiles. Notice M5 screws and washers. Hammer nuts are inside the extrusion profile grooves.

Fix both 22 cm extrusion profiles to 35 cm ones, and so on for all the other profiles as in Fig. 2. Connect the profiles using 2028 brackets, M5 screws, washers, and hammer nuts (Fig. 10).

Fix 25 cm to both long brackets connected to acrylic supports (Fig. 7, Fig. 8).

**Fig. 11:**
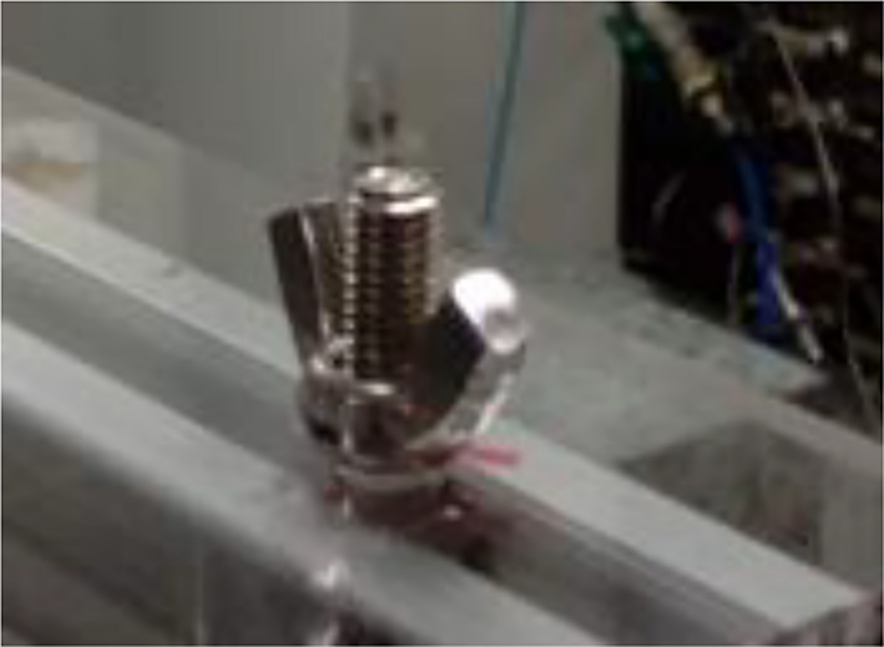
Acrylic sheet connected to extrusion profile using hammer-head screw and wingnut.

Fix the acrylic table to the extrusion profiles using M5 hammer-head screws (the heads go inside the grooves of the profiles) and wingnuts (Fig. 11).

**Fig. 12:**
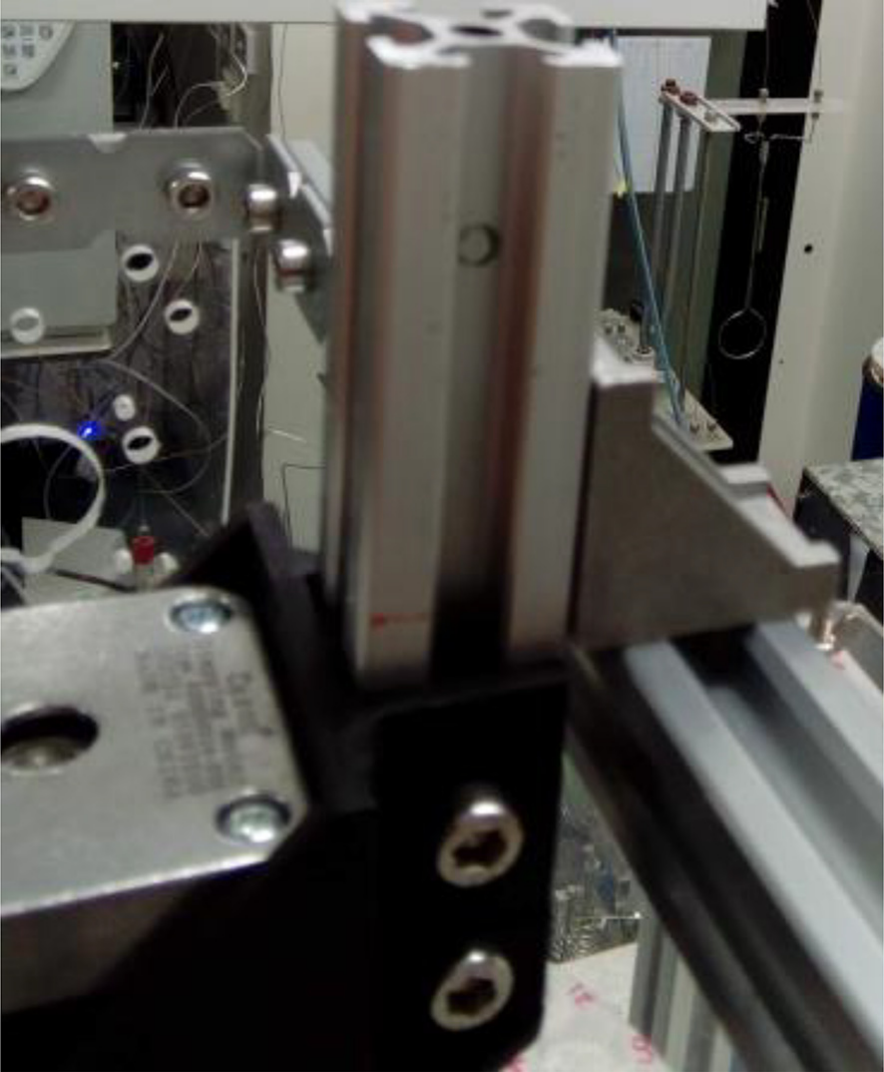
Stepper motor fixed to extrusion profile using stepper motor mount (Section 3).

Fix second stepper motor to the stepper motor mount (Section 3) using M3 screws, and the mount to a 10 cm extrusion profile using M5 screws, washers and hammer nuts (Fig. 12).

**Fig. 13:**
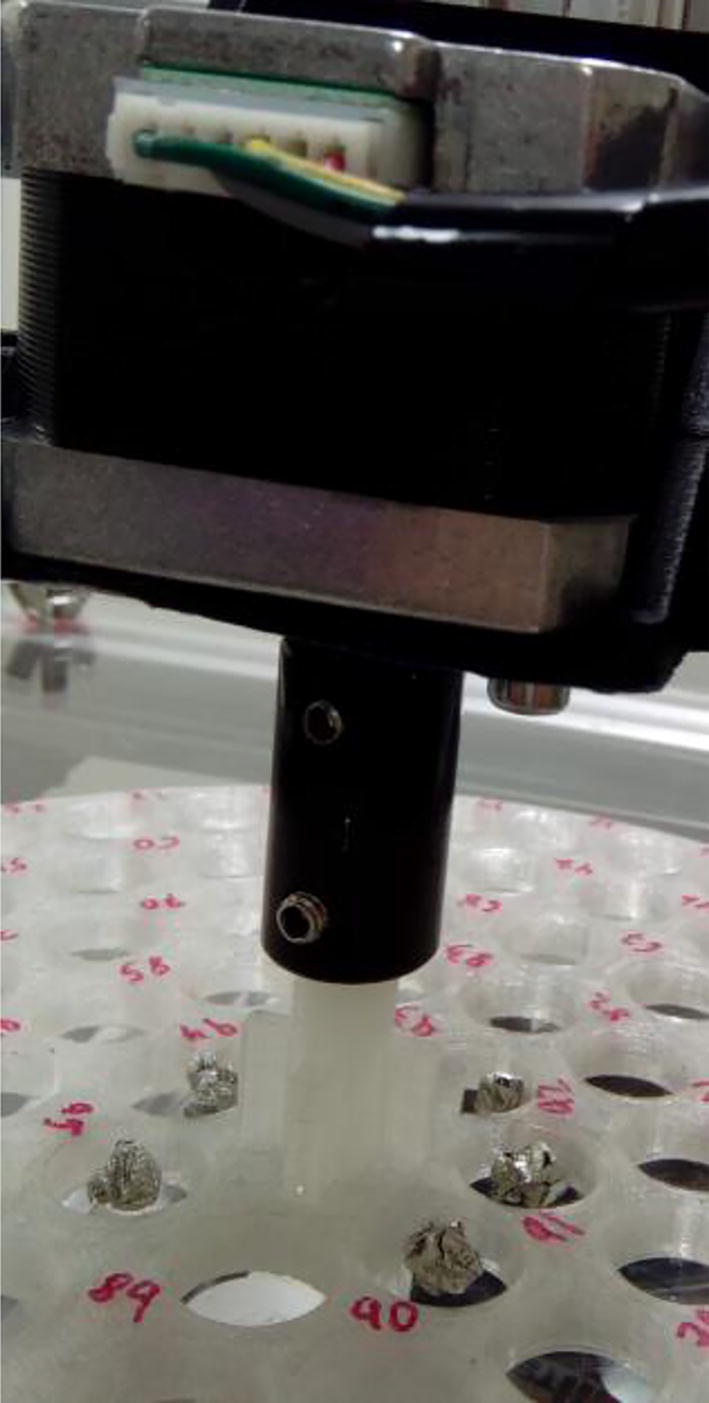
Carousel connected to stepper motor by shaft.

Connect carousel to stepper motor shaft (Fig. 13).

Finally, the carousel needs to be fixed to the gantry. This needs to be done so that the hole in the acrylic base is on the reach of each hole in the carousel. Also, the carousel must be close, but not touching, the acrylic. Place the stepper motor attached to the carousel on the acrylic sheet. At this point, they should already be connected to a 10 cm extrusion profile bar (Fig. 12). Place 3 sheets of normal printing paper between the carousel and the sheet, and then fix the 10 cm profile to the 25 cm profile (gantry between the two acrylic supports) using a 2028 bracket (Fig. 12). Remove the paper sheets. The table should be fully assembled by then.

### 5.3 Assembling the purging pipe

**Fig. 14:**
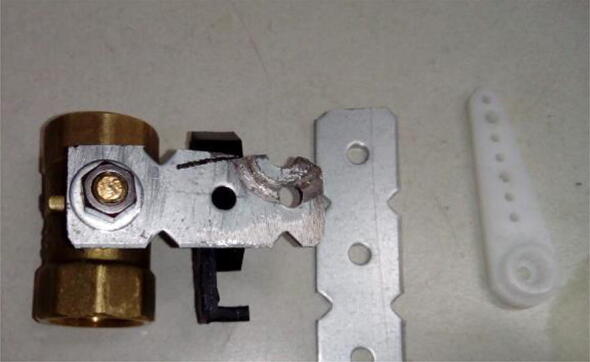
Valve with lever replaced by a custom-made one. This lever is made from a steel striping (middle of the figure), being cut and drilled according to the necessary dimensions. Here, the existing holes on the stripping were used as a starting point, and matching holes with enough diameter (here, 3 mm) were also drilled at the servo arm (right of the figure). The rotation center of the lever and the arm must also match when assembled (Fig. 15).

Remove the levers that come with the valves, and connect custom-made levers to the ball valves using the nuts that come with the valves (Fig. 14). Ensure that the levers make 90° with the main valve body when the valve is closed, and are parallel to the main valve axis when it is opened.

**Fig. 15:**
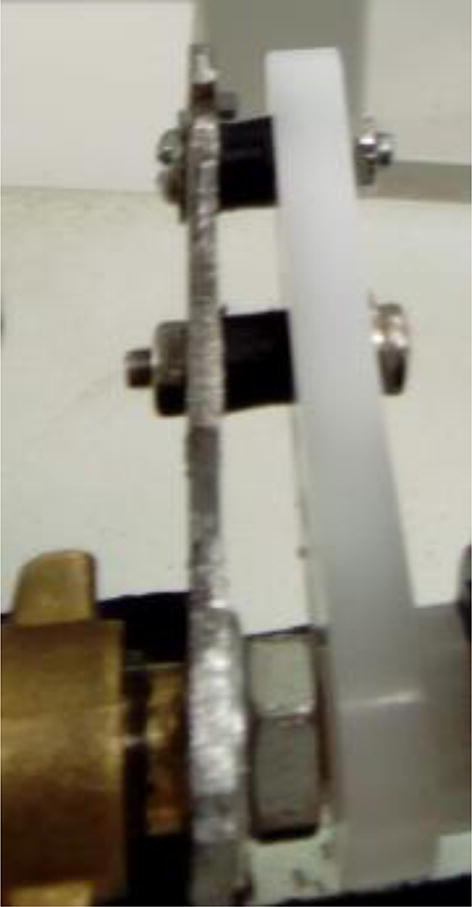
Valve lever connected to servo arm. Notice screws connected at opposing orientations.

Connect the servo arm to the lever using M3 screws, nuts and custom-made spacers listed in Section 3 (Fig. 15). Make sure that the M3 screws are connected at opposing orientations (Fig. 15). This ensures that the levers remain connected even after many repeated movements.

Before connecting the servo motor to its arm, make sure the shaft is at a known position that matches that of the arm-lever on the valve. For example, if the lever is making 90° with the valve, connect the servo at position 0°. The servo position can be setup using the control board, as described in Section 6.2.

**Fig. 16:**
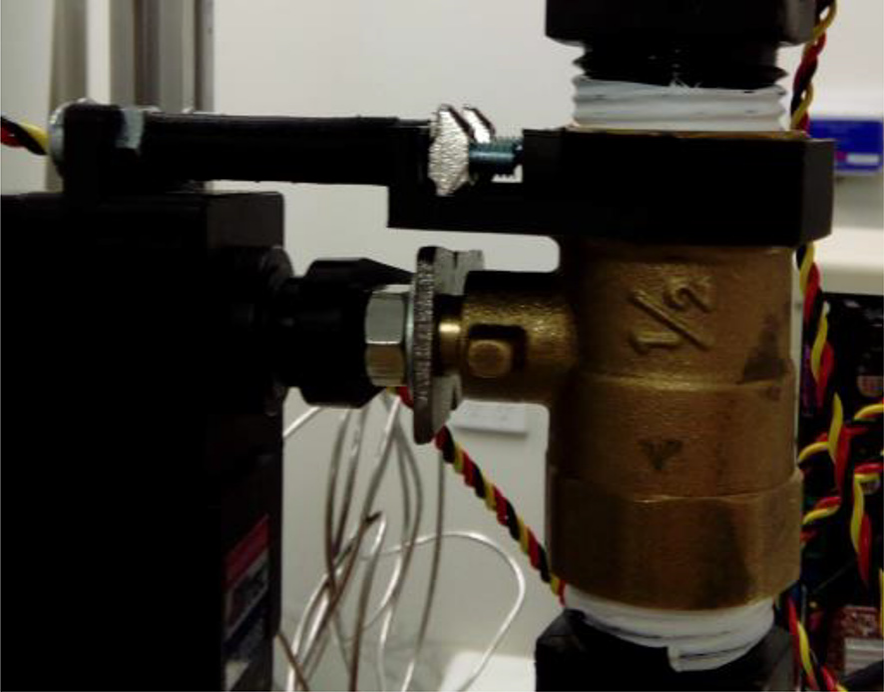
Servo motor fully connected to ball valve.

A second connection point between servo and valve is needed to make it possible for the servo to actuate the valve. Using M4 screws, nuts, washers, spacers (Section 3), and the servo-valve connector (Section 3), connect the servo to the valve (Fig. 16).

**Fig. 17:**
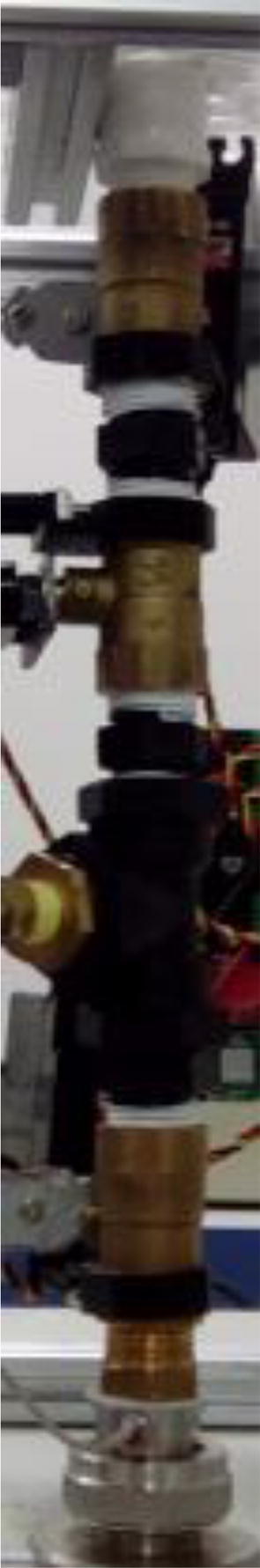
Purging pipe fully assembled.

Connect the valves to the remaining parts of the purging pipe (Fig. 17). Except for the topmost connection above the topmost valve, connections must be leak free. This can be ensured using either O-rings or Teflon tape. Gas leaks can be detected using appropriate devices. For helium, for example, a helium sniffer is very useful. For the topmost connection, use a connector which has no potential surfaces for samples to land on top and not fall to the surface of the valve. This is particularly important when analyzing small samples, which can potentially get trapped on such surfaces.

**Fig. 18:**
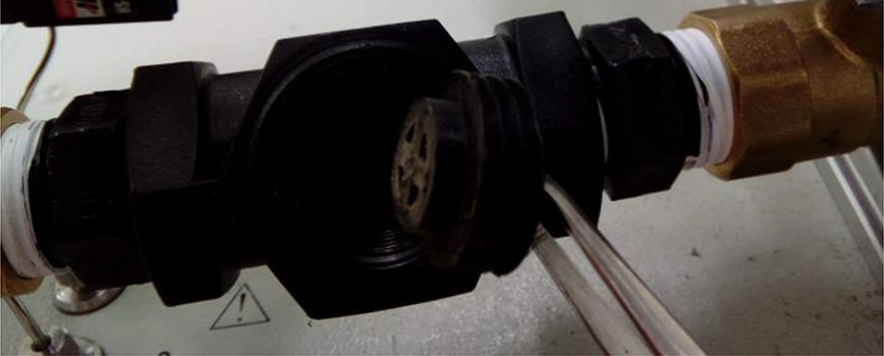
Sieve to be placed inside the T piece to avoid samples to settle at the perpendicular connection.

The connector between the lower and middle valve (a T piece) has a large dead space at the perpendicular connection. It is useful to add a sieve (Section 3) to avoid samples to settle at this part of the connector (Fig. 18).

Once fully assembled, connect the purging pipe to the elemental analyzer (Fig. 17).

### 5.4 Attaching the control boards and connecting the motors to the board

**Fig. 19:**
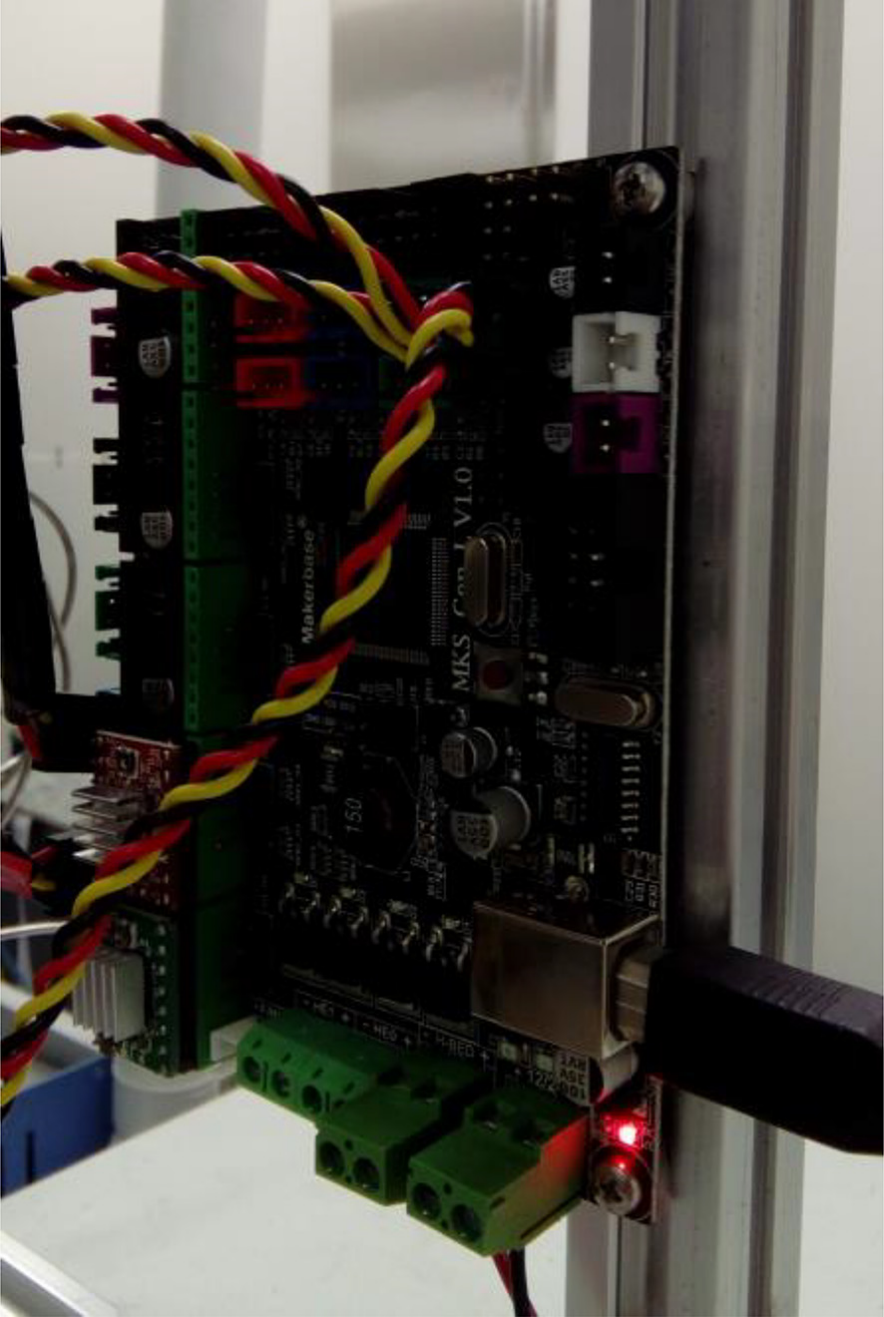
Control board fixed to the autosampler, positioned on reach of servo wires.

The control board is attached to one of the legs of the sampling table. The exact position must be decided when both the table and the purging pipe are placed on the elemental analyzer (Fig. 1), so that the wiring of the servo motors can reach the board without tension (Fig. 19).

**Fig. 20:**
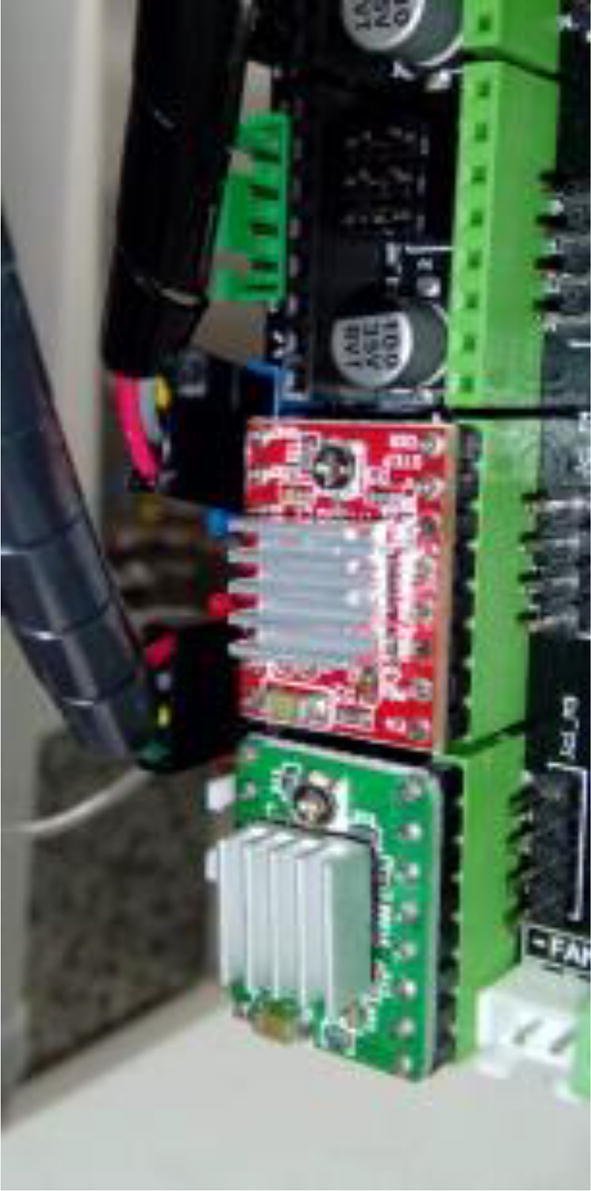
Stepper motor wires connected to the control board.

Connect stepper motors of each axis (X for gantry, and Y for carousel) to the appropriate stepper motor drive (Fig. 20). Importantly, connect the stepper motor drivers at the correct orientation (Fig. 20).

**Fig. 21:**
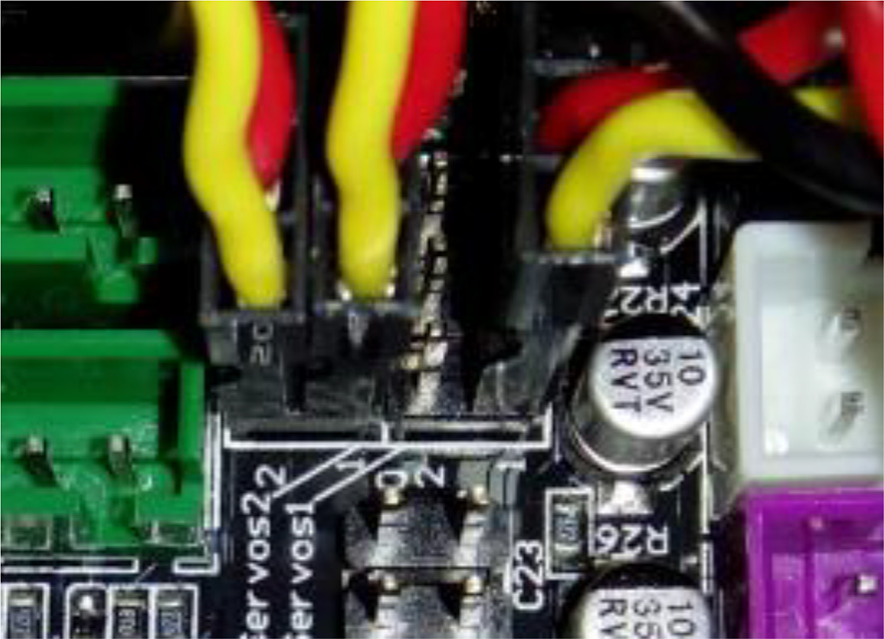
Servos connected to positions 0, 2 and 3. These positions are arbitrary and match the code in Supplementary Information 2. Different positions can be chosen, as long as the code is modified accordingly.

Connect servo motors to the appropriate slots (Fig. 21).

After motors are all wired to the board, the board can be powered with the power supply and connected to the computer using the USB cable (lower right corner of Fig. 21).

## 6 Operation instructions

### 6.1 Motor control

The firmware (OsmarMarlin, available from https://osf.io/qt9pg/ or https://doi.org/10.17605/OSF.IO/UTZSE) must be uploaded to the board using the Arudino IDE (available from https://www.arduino.cc/en/main/software). For this, the board must be connected to a computer via USB, and a BAUD rate of 115,200 should be used when uploading the firmware. This version of Marlin is an updated version of the one presented for previous autosamplers [29], [30], in order to become compatible with the current (at the time of writing) version of Arduino. On top of the modifications listed in these previous papers, two others were added to the configuration tab of this version to enable servo motor control: 1) the line “#define NUM_SERVOS” became #define NUM_SERVOS 4; 2) the line “#define SERVO_DELAY {3 0 0}” became #define SERVO_DELAY {300, 300, 300, 300}.

Motor control is done using G-code via the program Hype!terminal (available from https://osf.io/p7gh5/ or https://doi.org/10.17605/OSF.IO/UTZSE). The serial connection settings are: 115,200 for Baud rate, 8 data bits, none for parity, 1 stop bit, and none for flow control. The G-code dialect used is that for Marlin, thus including M commands [29]. The stepper motors are controlled using the G1 command: G1 <axis><distance><speed><movement speed>. For example, the command G1X10F500 means that the motor controlling the X axis will move 10 steps at 500 steps per minute. Importantly: before starting sending G commands, it is recommended that the command M121 is sent (just once after connection between board and computer is stablished), so that the full range of step values (including negative ones) can be reached.

Servo motors are actuated using the command M280, which has the usage M280P <motor number>S<angle in degrees>. The motor number can be 0–3, and the angle 0–180. For example, the command M280P0S130 will turn the shaft of the motor 0 (motor number corresponds to valve number, as explained before) to the angle 130° in relation to its origin.

For the carousel presented in Table 1 and Fig. 4, and for the stepper motors used here, we used feed rates of 50 (X axis) and 25 (Y axis). The X movement between carousel circles was found to be 1.1225. Therefore, to move from the initial circle (the most external one) to the next, the command was G1X1.1225F50. For the next, the step value was added of 1.1225, thus being G1X2.245F50. For the Y axis, the space between holes changes for each circle. For the most external the step was 0.09333, for the innermost 0.44167. Thus, for the most external circle the command from the first to the second hole was G1Y0.09333F25. If these values do not work for your setup, determine one by one by trial and error. For both X and Y axes, the best approach is to do a full movement (for example, from 0 to the maximum linear X range, or from 0 to the maximum Y circular range for each circle) and divide by the number of holes in each case.

**Table 1:** Comparison between the autosampler presented here and two commercial models.

Parameter	This autosampler	Costech Zero Blank Autosampler	Thermo-Fisher MAS200r
Number of samples per tray	95; can be expanded without modifying sample size if a larger tray is designed; or a larger tray can be built, along a larger table, so that a larger number of large samples can be accommodated	49 for 6 mm samples	32; more samples can be added if extra trays are pilled upon each other, but these need to be purchased separately
Maximum sample size	Sphere with 10 mm in diameter	Sphere with 6 mm in diameter when using a tray for 49 samples; it can take larger samples but then the tray needs to be swapped with one for less samples at once	Sphere with 10 mm in diameter, but only if purging flow is largely increased
Sample adding procedure	Samples need to be placed inside their respective holes in the carousel	Samples need to be placed inside carousel, and then a flushing procedure lasting about 10 min is needed. If more samples need to be added, the flushing needs to be repeated again.	Samples need to be placed inside their respective holes in carousel and a lid needs to be placed over the tray. If the lid is forgotten, large air blanks are introduced in the measurement.
Cost	AU$540 plus extras	AU$20,000	AU$10,000
Replacement parts	3D-printed or readily available from hardware stores	Proprietary	Proprietary
Compatibility	Universal via AutoIt	Proprietary, only for designed partner companies	Proprietary, only for designed partner companies

It is important to ensure that movement is consistent and reproducible. We have observed that if the control board is shut off and on again, the first movement of a stepper motor jitters. Thus, an adjustment is needed. This can be done using the command G92. For example, to determine that a given position is 0 for the Y axis, send G92Y0.

### 6.2 Integration with elemental analyzer

The autsosampler is synchronized to the elemental analyzer using AutoIt [24]. A script is written which coordinates commands between the software controlling the autosampler (Hype!terminal) and the software controlling the elemental analyzer (in this case, Isodat by Thermo Fisher). For a group of samples, the first sample to be measured is placed inside the purging pipe, directly on top of the top valve, which should be closed, as the other valves (Fig. 3). When isodat starts a measurement for the elemental analyzer, the status bar shows a message which AutoIt uses as a trigger for the autosampler. Once this trigger is received, the following actions take place: 1) the top valve opens, 2) the carousel spins (unless it is the first sample in a sequence, which will be already inside the purging pipe, as explained before), 3) the top valve closes; 4) the top valve and middle valve partially open, and remain opened for 60 s; 5) the top valve closes, and the middle valve opens, so that the sample falls on top of the bottom valve; 6) the middle valve closes; 7) the bottom valve opens, delivering the sample to the elemental analyzer; 8) the bottom valve closes, and the cycle can be repeated. The full code for all the steps is provided in Supplementary information 2.

The servo motors employed to actuate the purging pipe were capable of relatively high torque (1.94 N m). This torque demanded a relatively high current load which, if maintained for more than a few seconds (typically 60 s), caused problems of communication between the control board and the computer. This was dealt with by closing and opening communication between board and computer after at most three commands sent to the servos. This procedure cut the power supply and stopped servos from holding torque unnecessarily. The repeated execution of this procedure caused the COM port communication software (Hype!terminal) to crash, which was avoided by terminating and re-starting the program once every sample run. Despite considerable complexity being added to the operating of the autosampler, these steps ensured flawless sampling. A different combination of control board, communication software, ball valve and/or servo motor may prove more adequate and less demanding of computing and power resources, thus allowing a simpler operation of the system. Details of the procedure described here are given with the rest of the programming code in Supplementary Material 2.

### 6.3 Routine operation and maintenance

The most common procedure that demands partial disassembly of the autosampler is the removal of ashes from the elemental analyzer reactor, which must be done after a sufficient number of samples has been measured. It is not necessary to disconnect the servo motors from the board for this procedure, but, if it is decided to do so, it is advisable to firstly cut the power supply to the board. The purging pipe is then left at a safe position, and pushed along with the sampling table away from the top of the reactor. The reactor can now be pulled out of the furnace. The reactor can be hot, so care is needed during this operation. After ash removal, the reactor can be reinserted in the furnace, and the sampling pipe re-connected to it. Leaks need to be re-checked and, if none are found, the elemental analyzer can be re-started. If the power supply to the autosampler control board was cut, it must be re-connected, and the positions for the stepper motors verified, in particular the movement for the Y axis, which may jitter at the first execution.

It is advisable that sample remains are periodically cleaned from the autosampler. The carousel and its supporting table can be gently brushed after a sample run to remove small specs of samples, and occasionally rinsed with acetone if needed. Moving the carousel on its X and Y axes can help with the cleaning procedure. If this is done manually, it is necessary to re-set the positions of the axes on the controlling software to ensure proper operation.

It is good practice to always verify motor operation before starting a run to avoid simple accidents. This verification must always be done without any sample in the carousel.

If large samples are introduced in the autosampler, it is possible that they will get trapped by one of the ball valves and be destroyed. If this happens, it is possible that the valve becomes stiff and the servo cannot turn it properly. Disassembling the purging pipe and cleaning the valve is necessary in these cases. If the valve is still stiff after cleaning, it needs to be replaced, because continued operation of a stiff valve can ultimately damage the servo moving it. Therefore, it is good idea to have spares of these valves (and, in fact, of all components) for when such problems happen. If this kind of problem happens several times with the same valve, eventually the servo motor will also become damaged, and need to be replaced. Therefore, spare servos should be kept as well.

It is advisable to routinely (at least once every two weeks) inspect all screw connections in the purging pipe, and fasten the loose ones. Loose connections will result in incomplete valve movement. If the bottom valve does not close completely, a gradual increase in the N_2_ background from sample to sample, accompanied by a delay and broadening of sample peaks, are usually observed.

At the moment that the lower valve opens for the sample so that it falls into the reactor, the flow of the carrier gas is affected. If there is any blockage in the line, backpressure can push the contents of the reactor all the way to the top of the reactor, at its junction with the valve. This problem is avoided by keeping the gas lines unobstructed.

## 7 Validation and characterization

The autosampler was evaluated as an accessory for a Thermo Fisher Flash EA elemental analyzer coupled to a Thermo Fisher isotope ratio mass spectrometer. Samples were measured for N and C content using a modified Dumas combustion procedure [1], [2]. Samples were combusted under an oxygen pulse at 1020 °C in an oxidation reactor, where helium flowing at 150 mL min^−1^ carried the gases from combustion through chromium oxide pellets, and then through silvered cobalt ones. The gases were then transported to a reduction reactor kept at 650 °C and containing reduced copper wire pellets. The gas mixture was carried through a water trap (magnesium perchlorate), and then through a chromatographic column where gases were separated, notably N_2_ from CO_2_, because they are the gases being quantified for N and C content, respectively. Then the gases were carried through an interface (Conflo IV, by Thermo Fisher), where the gases were diluted so that they reached the mass spectrometer at 0.3 mL min^−1^. At the mass spectrometer, different protonated N_2_ (or CO_2_) isotopic configurations were deviated using a magnetic field and collected in faraday cups, where their relative abundances were quantified.

**Fig. 22:**
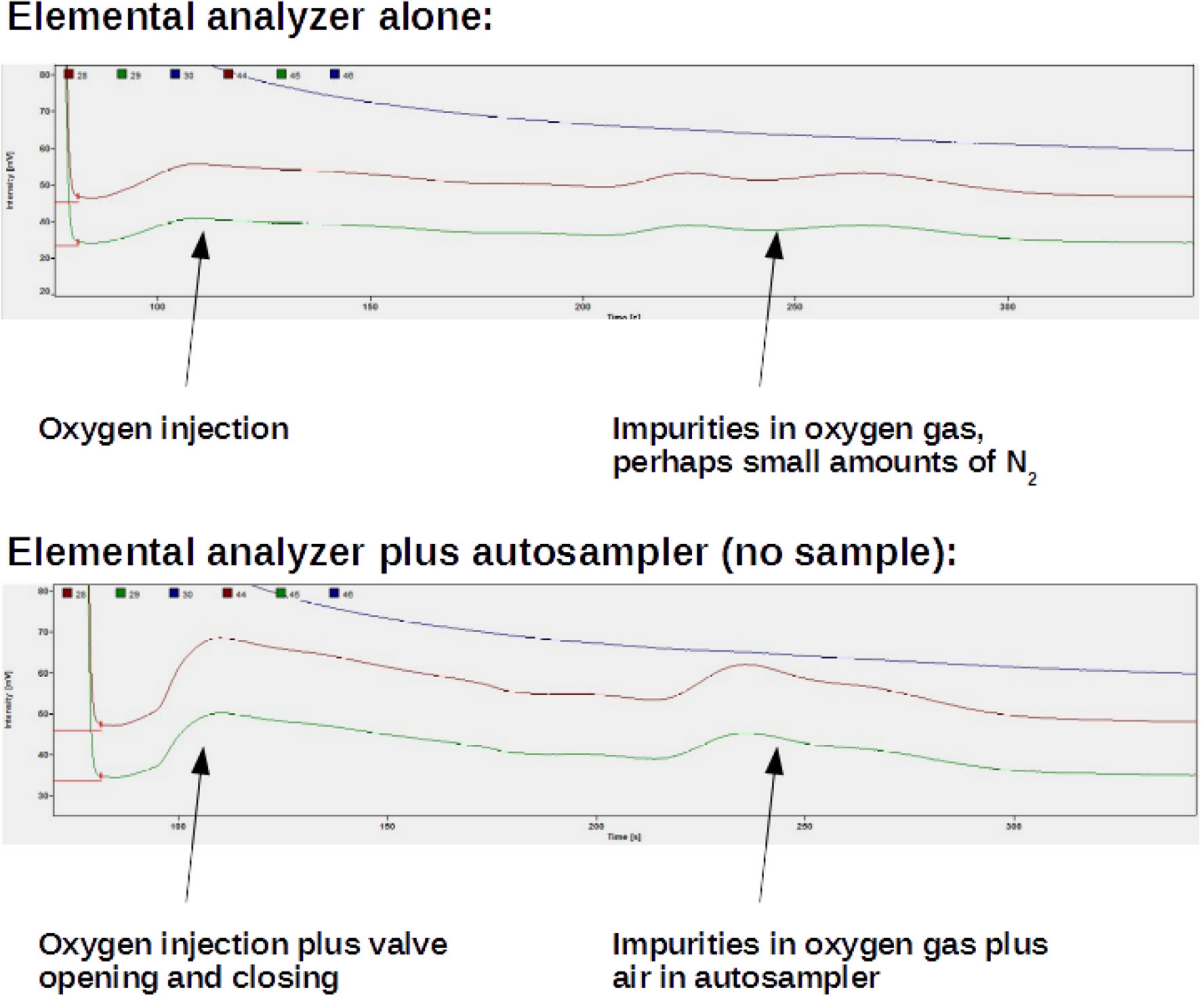
Comparison between chromatograms generated by elemental analyzer when only the elemental analyzer is used, and when the elemental analyzer is used together with the autosampler. Both chromatograms are shown with very large augmentation, and perturbations on the base line are very small in both cases.

**Fig. 23:**
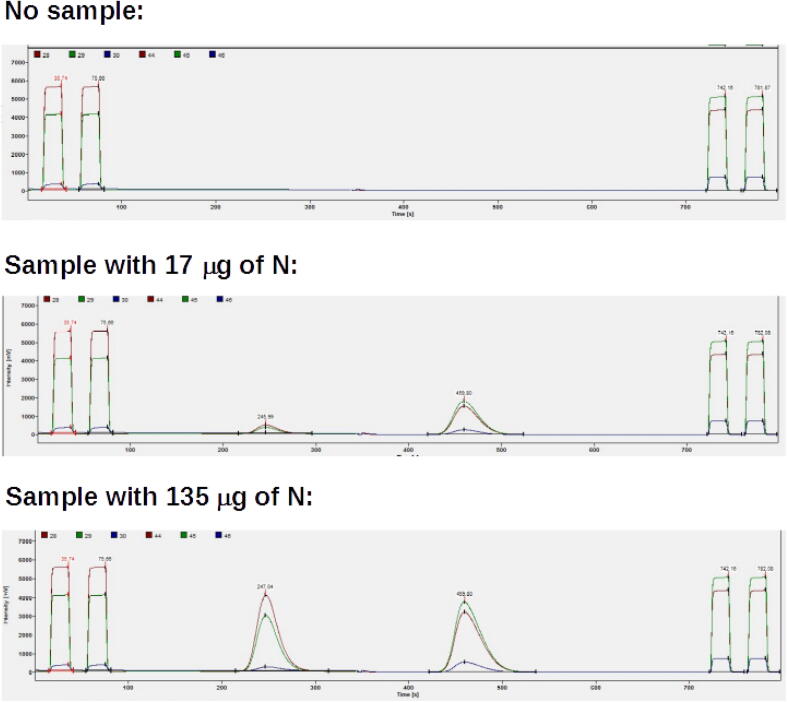
Comparison of chromatograms when the autosampler delivers no sample, and when samples with small and larger amounts of material are delivered. The first and last two peaks (the four rectangles) are reference gas peaks, the first two for N_2_, and the last two for CO_2_. The gaussian peaks are for the analyzed samples, the first one for N_2_, and the second one for CO_2_.

The main potential problem of the sampling procedure is air intrusion into the reactor together with the sample, which would interfere with measurements of gases similar to those abundant in the atmosphere, like N_2_ and O_2_. Thus, air input due to valve movement was evaluated by comparing it to operation of the elemental analyzer without any movement of the autosampler (Fig. 22). When used like that, the elemental analyzer modifies the flow by allowing an oxygen pulse into the helium stream, which slightly disturbs the baseline and adds a very small amount of N_2_, which is visible as a small peak latter in the chromatogram. When the autosampler was used, the perturbation of the baseline and the N_2_ peak were both slightly larger than when only the elemental analyzer was used (Fig. 22). However, such perturbations were very small in both cases, which is clear when the peaks from real samples are compared to the autosampler working with no sample (Fig. 23). There was also no effect on detector sensitivity, as samples with as little as 9 µg of N generated peaks with enough amplitude for useful isotopic measurements.

This test demonstrated that the autosampler fulfills the need for avoiding air contamination in the measurements. These results were obtained using the maximum flow for the purging gas (300 mL min^−1^) that the elemental analyzer permits. Even at this high flow rate helium consumption was low, because the valves are kept closed during most of the operation time.

Despite the negligible air influence on measurements, the valve movement caused a perturbation on the baseline (Fig. 22). This perturbation did not affect the sample peaks themselves, which came several seconds afterwards (Fig. 22). For isotopic measurements, which demand the measurement of reference gas peaks (the rectangular peaks in Fig. 23), valve opening was timed to happen only after these peaks for N_2_ had been finished in the chromatogram, thus ensuring no influence of valve movement on their measurement.

By the time this text was written, more than 6000 samples have been introduced in the elemental analyzer by the autosampler, generating useful scientific data. This has spanned more than a year of almost daily use of the autosampler. Routinely we have observed precision of 1% for both N and C contents, and 0.15‰ and 0.1‰ for N and C stable isotopes. These values are in accordance with the instrument specification, and demonstrate that the autosampler has not added significant errors to the measurements. Importantly, the autosampler was demonstrated solely for C and N analyzes of solids, but it could also be useful for other elements, like sulfur, oxygen and hydrogen, which are done using similar analytical equipment [31], [32], [33], [34], [1], [35].

The carousel employed here was designed with the specific needs of our laboratory in mind: it holds 95 samples for a single run, which allows almost 24 h of unassisted operation. Also, sample holes are wide enough to accommodate relatively large samples, which in our laboratory consist of sandy soils or sediments, and glass fiber filters of natural waters. More samples could be arranged for a single run, if so desired, by simply enlarging the carousel diameter, or making narrower sample holes. The provided 3D-printing file (Section 3) can be freely modified for such purposes.

The purging pipe was built using mostly of-the-shelf parts, but most of them could be replaced by custom-made, perhaps 3D-printed parts, so that the whole structure could be made shorter and simpler. These custom-made parts would need to be either made of metal, or of a heat-resistant, non-porous plastic in order to function properly.

Compared to commercial models (Costech Zero Blank Autosampler and Thermo Fisher A200s Autosampler), the model presented here has several advantages (Table 1). It is clear from there that the autosampler presented here is a low-cost and viable alternative to existing commercial models.

## 8 Human and animal rights

The work did not involve human or animal subjects.

## Declaration of Competing Interest

The authors declare that they have no known competing financial interests or personal relationships that could have appeared to influence the work reported in this paper.
