# Supplementary material for: Open-source autosampler for elemental and isotopic analyses of solids
Source: HardwareX. 2020 Jul 10;8:e00123. doi: 10.1016/j.ohx.2020.e00123 (PMC9041227; doi:10.1016/j.ohx.2020.e00123)
Supplement: Supplementary data 2 [file mmc2.docx]

**Explanation for AutoIt codes**

A basic knowledge of AutoIt or programming in general is assumed for the explanations given here. A complete and step by step introduction to AutoIt for laboratory automation can be found elsewhere[1].

**List 1:** **AutoIt code integrating Hype!terminal (the software controlling the autosampler) and Isodat (the software controlling the elemental analyser).**

#RequireAdmin

#include <AutoItConstants.au3>

Opt("WinTitleMatchMode",1)

$Hypecontrol = "WindowsForms10.EDIT.app.0.141b42a_r12_ad11"

Dim $Holesy[5]

$Holesy[0]=34

$Holesy[1]=24

$Holesy[2]=17

$Holesy[3]=13

$Holesy[4]=7

Dim $Stepy[5]

$Stepy[0]=0.0933

$Stepy[1]=0.1335

$Stepy[2]=0.186875

$Stepy[3]=0.23917

$Stepy[4]=0.4416666666666667

$turn = 0

For $x = 0 to 4

Move("Y",0,25,10000,$Hypecontrol)

Move("X",1.1225*$x,50,2000,$Hypecontrol)

For $y = 1 to $Holesy[$x]

$turn = $turn+1

ConsoleWrite($turn&" "&@HOUR&@MIN&@SEC&@CRLF)

WaitIsodatStatusBar("Event - 2 s: Trigger All ConFlo IV/Ext Trig 2a -> Off [2 s]")

ServoMove(2,90,1000,$Hypecontrol,2)*;valve 2 open*

Move("Y",$Stepy[$x]*($y-1),25,1000,$Hypecontrol)

ManyServo()

Sleep(60*1000)

OffOnComplete($x*1.1225,$Stepy[$x]*($y-1),12000,$Hypecontrol)

Next

Next

*; Functions for basic moves below*

Func Move($direction,$position,$speed,$wait_time,$control)

ControlSetText("Hype!","",$control,"G1"&$direction&$position&"F"&$speed)

Sleep(1000)

ControlSend("Hype!","",$control,"{ENTER}")

Sleep($wait_time)

If WinExists("HyperTerminal-CM") Then Exit

EndFunc

Func ServoMove($number,$position,$wait_time,$control,$turns)

For $i = 1 to $turns

ControlSetText("Hype!","",$control,"M280P"&$number&"S"&$position)

Sleep(500)

ControlSend("Hype!","",$control,"{ENTER}")

Next

Sleep($wait_time)

If WinExists("HyperTerminal-CM") Then Exit

EndFunc

*; Composite functions below*

Func OffOnSimple($wait_time)

BlockInput($BI_DISABLE)

WinActivate("Port_Configuration")

WinMove("Port_Configuration","",0,0)

MouseClick("left",250,310,2)

Sleep(1000)

MouseClick("left",300,90,2)

BlockInput($BI_ENABLE)

Sleep($wait_time)

EndFunc

Func OffOnComplete($positionX, $positionY, $wait_time,$control)

BlockInput($BI_DISABLE)

WinClose("Hype")

WinWaitClose("Hype")

Sleep(1000)

Run("C:\...\Desktop\Hype!Terminal.exe")

While Not WinExists("Hype")

Sleep(100)

WEnd

WinActivate("Hype!")

Sleep(1000)

Send("!c")

Sleep(1000)

Send("{TAB 2}")

Sleep(1000)

Send("{DOWN 5}")

Sleep(1000)

Send("{TAB 5}")

Sleep(1000)

Send("{DOWN 2}")

Sleep(1000)

Send("{TAB 1}")

Sleep(1000)

Send("{SPACE}")

Sleep(2000)

BlockInput($BI_ENABLE)

ControlSetText("Hype!","",$control,"M121")

Sleep(1000)

ControlSend("Hype!","",$control,"{ENTER}")

Sleep(1000)

ControlSetText("Hype!","",$control,"G92X"&$positionX&"Y"&$positionY)

Sleep(1000)

ControlSend("Hype!","",$control,"{ENTER}")

Sleep($wait_time)

EndFunc

Func ManyServo()

ServoMove(2,0,1000,$Hypecontrol,2)*;valve 2 closed*

ServoMove(2,30,1000,$Hypecontrol,2)*;valve 2 half open*

ServoMove(3,70,5000,$Hypecontrol,2)*;valve 1 half open, purge*

OffOnSimple(60000)

ServoMove(2,0,2000,$Hypecontrol,2)*;valve 2 closed*

ServoMove(3,120,500,$Hypecontrol,2)*;valve 1 open*

OffOnSimple(500)

ServoMove(3,35,500,$Hypecontrol,2)*;valve 1 closed*

OffOnSimple(500)

ServoMove(0,100,500,$Hypecontrol,2)*;valve 0 open*

ServoMove(0,0,500,$Hypecontrol,2)*;valve 0 closed*

EndFunc

Func WaitIsodatStatusBar($message)

$equal = False

While $equal = False

$status1 = ControlGetText("Isodat Acquisition","","Static61")

$status2 = ControlGetText("Isodat Acquisition","","Static6")

If $message = $status1 OR $message = $status2 Then

$equal = True

Else

Sleep(100)

EndIf

WEnd

EndFunc

The code has two main sections: main code (lines 1 to 36) and functions (lines 38 to 132). We will start with the functions.

**Explanation of functions**

The first two functions (lines 40 to 56) are used to control the motors in the autosampler via Hype!terminal. Function “Move” controls the two stepper motors (axes X and Y), while ServoMove controls the three servo motors.

The function Move calls the command ControlSetText to set the text of the input field on Hype!terminal (Fig. S2.1). ControlSetText receives the following arguments: window title (in this case, the word “Hype”, window text (left blank, indicated by “”), the control to be modified (indicated by the variable $control), and the text to be set (the remaining of the parameters inside brackets, including the variables $direction, $position, and $speed).


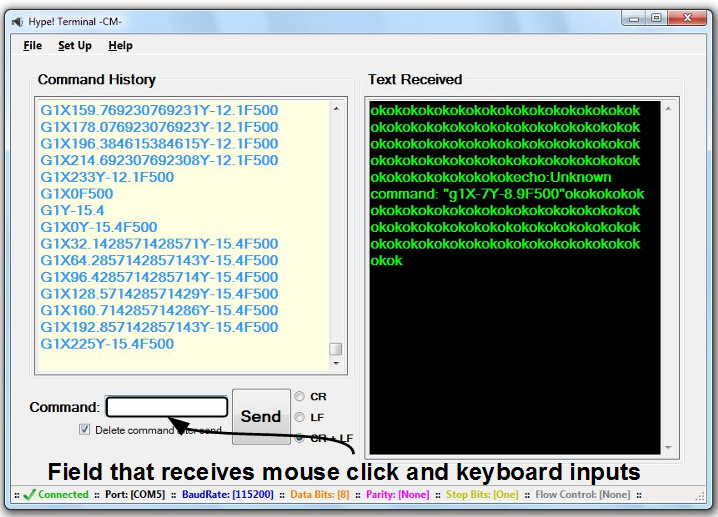


Fig. S2.1. Hype! Terminal interface with the input field highlighted.

A waiting time of 1 s (indicated by the Sleep function in line 42) is given, then the command ControlSend is called, with similar arguments to the previous ControlSetText function, except that the text to be sent is resumed to the Enter key. We observed that using a single ControlSend command would not work properly on some computers, and therefore divided the instruction in two, which has worked flawlessly since then.

The next instruction (line 44) is another waiting time, which is determined by the variable $wait_time.

The final line in the function (line 46) is a safety instruction to tackle a fail in the communication that results in the displaying of the window titled “Hyperterminal-CM” (Fig. S2.2) by the Hype!terminal program.


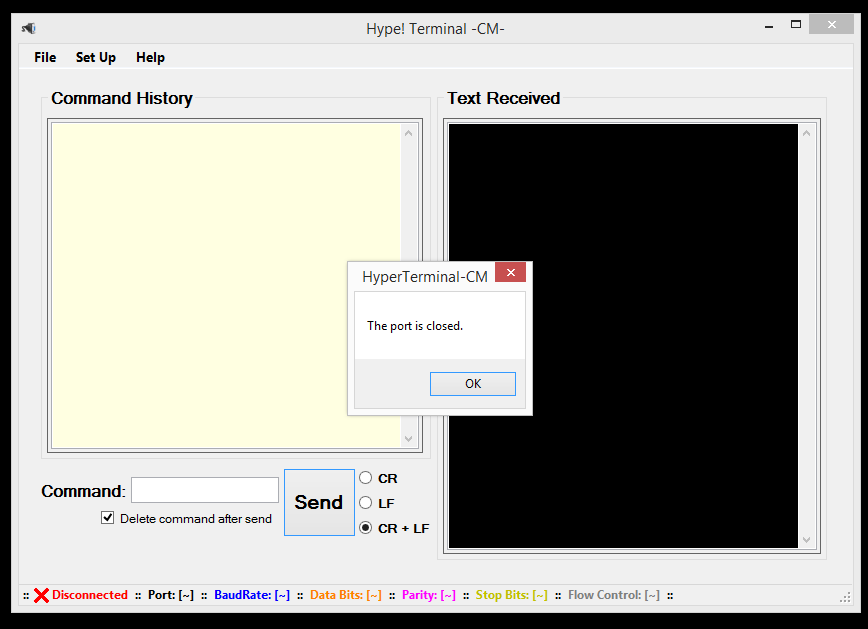


Fig. S2.2: Window indicating communication fail between computer and control board.

The next function, ServoMove, is very similar to Move. The main differences are the arguments being passed to it, which now include: $number, which is equivalent to $direction (we call it differently here because for steppers usually direction corresponds to one of the axes, while for servos are simply listed from 0 to 3); $position; does not include $speed; $wait_time; $control; and $turns, which is the number of times that a given instruction is sent to the board every time the function is called. We decided to include this last variable because in initial tests we found that the servos would, even if very rarely, fail to respond to a command. By sending the command repeatedly we found that servos would never fail to move, and that this would also not increase the execution time of the script if the Sleep intervals were kept short. Thus, in the function there is a for loop which calls the commands ControlSetText and ControlSend as many times as determined by the variable $turns.

The next function (OffOnSimple) closes and opens the communication between computer and board by means of some mouse clicks sent to the “Port_Configuration” window (Fig. S2.3).


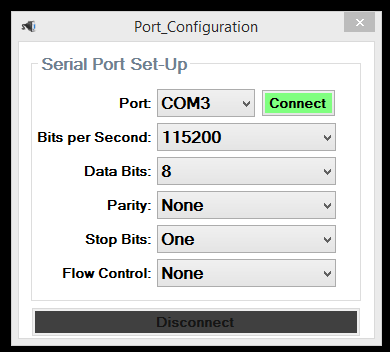


Fig. S2. 3: Port_Configuration window.

Port_Configuration is a sub-window of the program Hype!terminal, which can be accessed from the Setup menu (Fig. S2.1). Port_Configuration must obviously be open when the OffOnSimple function is called. So, throughout the execution of the script, this window must be left open. The first command in the function is BlockInput with the parameter $BI_DISABLE, which locks the mouse and keyboard. This is a safety measure to ensure that the computer user will not interfere with script execution. Then, the window is activated (WinActivate) and moved (WinMove) to a fixed position. Two mouse clicks, called using the function MouseClick, are sent with a 1 s space in between them. The first click is on the “Disconnect” button, the second one on the “Connect” button (Fig. S2.3). BlockInput is called again, but this time with the parameter $BI_ENABLE, which restores the user ability to control the mouse and keyboard. The final command is to wait for the time passed as an argument for the function. In theory, it would have been possible to define this function using direct access to controls (using the command ContrlClick) instead of relying on direct mouse clicks. However, our experience has been that it is more reliable to rely on mouse clicks in this case, and thus we have chosen this option.

The next function, OffOnComplete, does not simply close and open communication between computer and control board, it closes the Hype!terminal program, opens it again, restores communication, and resets positions for axes X and Y, because once communication is closed these positions are lost, and these positions are necessary for the remaining of the execution of the script. This function is called once every sample run in order to prevent fail by the Hype!terminal program (we have observed that if the OffOnSimple function is called repeatedly many times, the program crashes).

As for OffOnSimple, OffOnComplete starts by blocking computer access (BlockInput), then it closes the Hype!terminal window (WinClose). The next command (WinWaitClose) ensures that the script will only proceed after the window is closed. A waiting time of 1 s is given, then Hype!terminal is re-started using the command “Run”, which takes as argument the complete path to the program’s executable file (this needs to be determined on each computer). The line is a while loop using the command WinExists for the Hype!terminal window. The loop checks every 0.1 s until the window is open. After that, the window is activated (WinActivate), and a sequence of key strokes are sent. The first one is “!c”, which means the Alt key followed by the c key. This combination opens the Port_Configuration window. After that, the Tab key is sent twice, so that the field identified as “Bits per second” (Fig. S2.3) is highlighted. Then the down arrow key is sent five times so that the value 115200 is chosen. The tab key is sent another 5 times, so that the “Port” field is selected. Then the down arrow key is sent twice (this number can be different depending on the actual port being used by the control board). The Tab key is sent once more, to select the button “Connect”, and then the Space key is sent to activate the button. The next command is to restore access to the computer (BlockInput).

The final commands in the function send texts to the Hype!terminal window using controls, and not direct keyboard entries, by means of the functions ControlSetText and ControlSend, as done in the functions Move and ServoMove, previously presented. The first text is “M121”, which enables the use of negative numbers for G1 commands (this is not necessary in the present code, but it is useful if the script is paused and the autosampler position needs to be reset). The second text is the command G92 followed by current X and Y positions. These positions are given as arguments for the function.

The function ManyServo is simply a sequence of many ServoMove functions interspaced with OffOnSimple functions. Each servo movement is described as a comment in the function. The long waiting time at line 111 was used in order to properly synchronize sample input by the autosampler and oxygen injection by the elemental analyser. This kind of fine tuning needs to be determined for each case, and may vary between different kinds of analysers and software.

The function WaitIsodatStatusBar checks the status bar of the Isodat program (Fig. S2. 4). The variable $equal gets the False value, then a While loop keeps looping while this variable has this value. The value only changes when either the variable $status1 or the variable $status2 becomes equal to the variable $message, which is the argument for the function. Both $status1 and $status2 refer to the message displayed at the status bar of the Isodat software. The status bar is checked every 0.1 s.


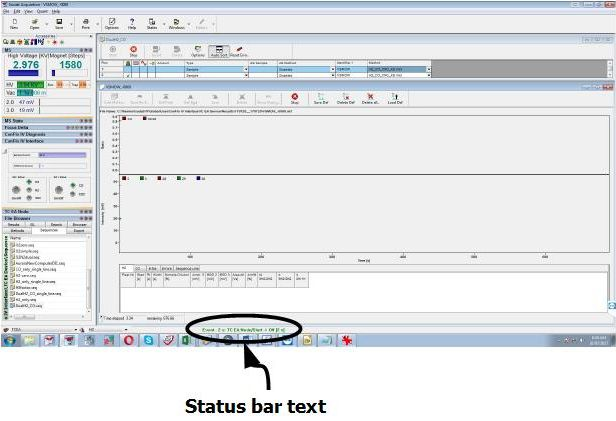


Fig. S2.4: Isodat with its status bar highlighted.

**Explanation of the main code**

**Main code**

Now that the functions have all been described, we can examine the main part of the script (lines 1 to 36). The first line enables the script to be executed with administrator privileges. This is necessary for the command BlockInput, called at some parts of the script. The next line includes some variables, specifically $BI_DISABLE and $BI_ENABLE, both necessary arguments for the command BlockInput. The third line enables the use of only the initial portion of a window name as argument in commands.

The next line defines the variable $Hypecontrol, which refers to the input field in the Hype!terminal window (Fig. S2.1). The information about a control, as shown here, can be obtained using the utility AutoItWindowsInfo[1].

The next two sections of the script (lines 7 to 12 and 14 to 19) declare two arrays. $Holesy is an array for which each element contains the number of holes on each circle of the carousel (Fig. 4, in the main text; see also the file provided in Table 3, main text): the most external circle has 34 holes, the next one 24, and so on, until the innermost circle, which has only 7 holes. The next array, $Stepy, contains the Y step between two holes in each circle. This step is 0.0933 units for the outermost circle, and increases for each circle, becoming 0.44167 for the innermost one.

Subsequently, the variable $turn is declared as being 0. This is a counter to allow the monitoring of the operation of the autosampler, as it will be shown next.

The main part of the script is the for loop between lines 23 and 36. The variable $x refers to the five concentric circles in the sample carousel, numbered from 0 to 4. Two Move instructions are sent, the first bringing the carousel to position Y = 0, and the next to position X = 1.1225 times $x. This means that for each step of this loop the carousel will be placed at the initial hole of one of the concentric circles. The following loop, from lines 27 to 35, spins the carousel through each loop. This second loop as a variable size, determined by the value of the variable $Holes[$x], corresponding to the number of holes in the circle, as previously explained. The variable $turn is incremented of a step, and it is printed, along the time of sampling, on the console of the SciTE editor (Fig. S2.5). Then, the script waits for the message on Isodat (WaitIsodatStatusBar), opens the topmost valve and moves the carousel one step. Then the function ManyServo is called, a waiting time of 60 seconds is given, and the function OffOnComplete is called. The loop repeats until the last sample, unless the script is stopped beforehand. On some computers, this can’t be done at SciTE interface, but must be done by forcing the script to stop. This can be done by accessing the script from the right-down corner, and choosing Exit after a right click on its icon (Fig. S2.6).


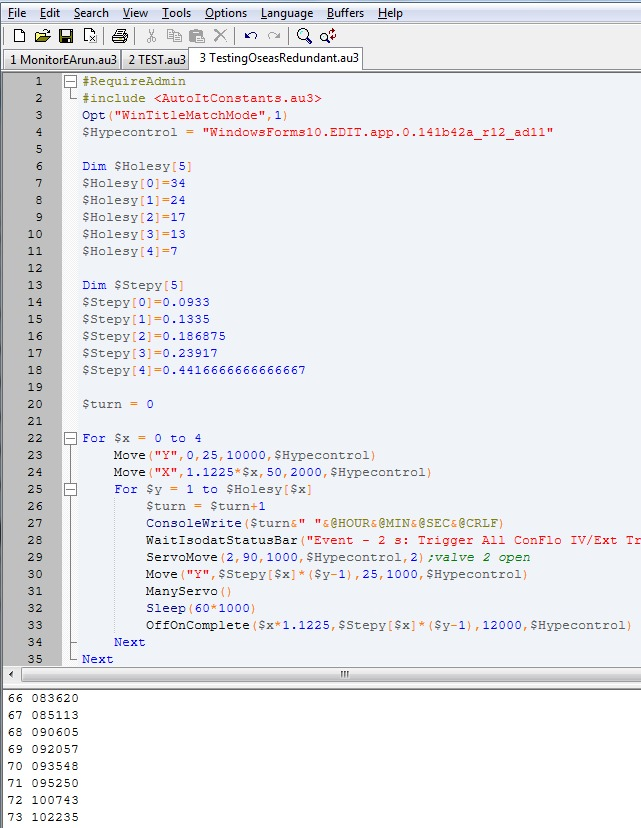


Fig. S2. 5: SciTE editor window with console (lower portion of the window) displaying the value of the variable $turn (66 to 73), followed by hour (two digits), minute (two digits) and second (two digits), all combined as a single number.


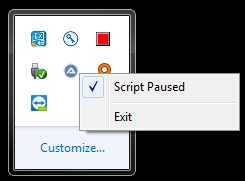


Fig. S2.6: Right-down corner menu showing the script icon (central icon) and the menu that appears when it receives a right click. Exit should be chosen in order to properly end the script on some computers.

[1] M.C. Carvalho, Practical laboratory automation made easy with AutoIt. Wiley VCH, (2016)
